# A Comprehensive Targeted Metabolomics Assay for Crop Plant Sample Analysis

**DOI:** 10.3390/metabo11050303

**Published:** 2021-05-11

**Authors:** Jiamin Zheng, Mathew Johnson, Rupasri Mandal, David S. Wishart

**Affiliations:** 1 Departments of Biological Sciences, University of Alberta, Edmonton, AB T6G 2E9, Canada; jiamin3@ualberta.ca (J.Z.); mkj1@ualberta.ca (M.J.); rmandal@ualberta.ca (R.M.); 2 Departments of Computing Science, University of Alberta, Edmonton, AB T6G 2E9, Canada

**Keywords:** crop plant, conifer needles, cannabis buds, canola roots, plant metabolomics, LC-MS/MS

## Abstract

Metabolomics plays an important role in various fields from health to agriculture. However, the comprehensive quantitative metabolomic analysis of plants and plant metabolites has not been widely performed. Liquid chromatography coupled with tandem mass spectrometry (LC-MS/MS)-based plant metabolomics offers the sensitivity and breadth of coverage for both phenotyping and disease diagnosis of plants. Here, we report a high-coverage and quantitative MS-based assay for plant metabolite analysis. The assay detects and quantifies 206 primary and secondary plant metabolites, including many key plant hormones. In total, it measures 28 amino acids and derivatives, 27 organic acids, 20 biogenic amines and derivatives, 40 acylcarnitines, 90 phospholipids and C-6 sugars. All the analysis methods in this assay are based on LC-MS/MS techniques using both positive and negative-mode multiple reaction monitoring (MRM). The recovery rates of spiked plant samples at three different concentration levels (low, medium and high) ranged from 80% to 120%, with satisfactory precision values of less than 20%. This targeted plant metabolomic assay has been successfully applied to the analysis of large numbers of pine and spruce needle samples, canola root samples, as well as cannabis samples. Moreover, the assay was specifically developed in a 96-well plate format, which enables automated, high-throughput sample analysis. This assay has already been used to analyze over 1500 crop plant samples in less than two months.

## 1. Introduction

Plants exhibit extraordinary chemical diversity. In fact, it is estimated that the plant kingdom, as a whole, likely produces more than 600,000 metabolites [1]. This chemical diversity is a consequence of the fact that plants are not motile. To survive attacks from pathogens and insects or thrive under conditions of drought or flooding, plants must defend themselves not by running away, but through targeted chemical warfare. In other words, plants must produce and use specific chemicals to assist in defending against insect herbivory, in fighting against bacteria and viruses, in responding to heat and drought, or in battling against cold and frost. The rich chemical response that plants have to battle biotic and abiotic stress has drawn many plant scientists to study the plant metabolome. In the field of plant studies, metabolite profiling has been widely used not only to study stress response in plants but to assist with gene annotation [2], to perform plant phenotyping or chemotyping [3,4,5], to assist with disease detection or analysis [6] and to discover new medicinal compounds [7,8,9,10]. The chemicals identified through these studies include both primary and secondary plant metabolites. Primary metabolites, such as amino acids, carbohydrates, and organic acids, reveal how plants change their general metabolism to adjust, respond and continue to grow under different conditions. Secondary metabolites, such as phytosteroids, alkaloids, terpenes, and various plant hormones such as abscisic acid, salicylic acid and jasmonic acid, have been studied to better understand the signaling events that occur between plants and their environment.

Due to the wide chemical diversity as well as the enormous concentration ranges of plant primary and secondary metabolites (from femtomolar to millimolar), a number of different analytical platforms must be used to study plant metabolomes. These include NMR [11,12,13], CE-MS [14], GC-MS [15,16,17], and LC-MS [18,19,20]. NMR-based metabolomics is particularly useful for quantifying metabolites and for detecting hydrophilic compounds such as sugars, alcohols and organic acids [11,12,13], but the poor sensitivity of NMR (with a lower limit of detection of ~5 µM) has limited its use. In contrast, the higher sensitivity of LC-MS, CE-MS and GC-MS techniques has allowed plant researchers to cover a much wider swath of the plant metabolome. For instance, 53–148 metabolites have been identified and quantified in rice, tobacco and the rosy periwinkle using CE-MS and CE-TOF-MS [14,21,22,23]. However, CE-MS assays can be quite difficult to develop and the compounds detected are generally limited to charged compounds. On the other hand, GC-MS-based metabolomic methods are much simpler to perform and are not quite so limited in their molecular coverage. GC-MS-based metabolomics has been particularly useful in detecting and quantifying lower molecular weight primary metabolites (amino acids, sugars, fatty acids and sugar alcohols) in a number of plant species [23,24,25,26], with up to 126 compounds being detected in tobacco leaves [23]. GC-MS has also been used to identify and quantify plant secondary metabolites, which tend to be present in very low concentrations in plant tissue. To expand the coverage of detectable secondary plant metabolites by GC-MS, a number of very useful derivatization protocols have been developed [27,28,29]. GC-MS has also been applied to identify volatile compounds, such as terpenes and other scent-related compounds in fruits, vegetables and flowers [30,31,32]. Unfortunately, the throughput of GC-MS methods is relatively low and the coverage of higher molecular weight compounds, such as lipids, waxes, polyphenolic glycosides and phytosteroids, is limited.

Currently, the most popular platform in plant metabolomics is LC-MS. This is because LC-MS offers the speed, the sensitivity, the wide molecular weight window and the breadth of coverage needed to analyze the myriad of plant metabolites whose concentrations can vary by several orders of magnitude. To date, most of the LC-MS assays that have been developed for plant metabolomics studies have been of the untargeted variety [19,33,34,35]. Some of these untargeted assays have been able to detect up to 857 metabolites in corn [36], up to 350 metabolites in tomatoes [37] and up to 286 metabolites in grain [36]. While untargeted assays offer impressively broad coverage, they do not provide accurate quantification. To accurately quantify metabolites by LC-MS, one must use targeted assays with isotopically labeled standards. A thorough review of the literature has revealed that there have been surprisingly few targeted plant metabolomic assays developed or described. The majority of targeted LC-MS assays could detect and quantify only one or two metabolite classes [38,39,40]. Among the most extensive targeted assays that we could identify were those that could detect and quantify up to 393 plant lipids [41], 28 plant polyphenols [42], and 51 primary metabolites [20]. Among the targeted assays that we identified, it was apparent that most were not designed for high-throughput analysis and that none could really combine the detection of both hydrophobic (i.e., lipids) and hydrophilic compounds (sugars, amino acids, etc.). This suggested to us that there is a need to develop a comprehensive, targeted, high-throughput LC-MS assay that could detect and quantify large numbers (>200) of diverse primary and secondary plant metabolites.

Previously, we have described the development and implementation of targeted LC-MS assays to analyze mammalian biofluids such as urine [43] and serum/plasma [44]. However, mammalian metabolomes are very different than plant metabolomes. For instance, the concentrations of amino acids such as glutamine and glutamate are very different and there are large numbers of organic acids, such as abscisic acid, salicylic acid and jasmonic acid, that are not found in mammalian samples. Furthermore, the tissues and cell structures of plants are very different from mammalian tissues or biofluids. Therefore, assays developed for mammalian biofluids or tissues cannot be directly applied to plant tissue samples. In order to develop a proper plant assay that could work for most plant samples, it was necessary to not only substantially modify our existing targeted assays, but also to develop an optimized extraction workflow for plant tissues to find and test many new isotopic standards, and to recalibrate all reference concentrations. Through these changes, we were able to develop a comprehensive targeted LC-MS/MS assay that can absolutely quantify up to 206 primary and secondary plant metabolites. The assay can detect many key plant hormones as well as amino acids, amino acid derivatives, biogenic amines, organic acids, sugars, acyl-carnitines, and phospholipids. The assay was specifically developed to accommodate the wide range of compound concentrations found in different plant tissues and to manage the very different values seen in plant metabolomes relative to mammalian metabolomes. To demonstrate the utility of this assay, we show how it can be used to accurately analyze different plant tissues (roots, flowers and leaves) from different crop species, ranging from annuals (canola, cannabis) to seed/nut bearing trees (pine, which produces pine nuts, and spruce). As we were able to identify and quantify metabolites from such diverse plant species and plant tissues, we are confident that this assay could also be used to analyze fruits, vegetables, nuts and seeds. Furthermore, this plant-specific assay was developed to facilitate high-throughput analysis, allowing up to 82 samples to be run in a 45-h period on a single properly equipped LC-MS instrument.

## 2. Results and Discussion

### 2.1. Optimization of Extraction of Metabolites from NIST Standard Reference Material (SRM)

The extraction of metabolites from plant samples should be comprehensive to cover as many different classes of metabolites as possible, including both hydrophilic and lipophilic metabolites. To maximize the recovery of these different classes of metabolites, we tested different solvents and different sample-to-solvent ratios to extract metabolites. As there are relatively few traditional crop plant reference materials available, the standard reference material (SRM) we decided to use was the NIST^®^ SRM^®^ 1575a for pine needles. Note that pine trees produce edible pine nuts and so they are considered a crop plant/tree in Canada and elsewhere.

No single solvent can extract all the hydrophobic and hydrophobic metabolites of a whole plant metabolome. Therefore, a solvent system that combines both the polar and non-polar solvents will more likely extract both types of plant metabolites. The solvent system or liquid–liquid extraction allows the partitioning of metabolites into two immiscible phases, aqueous and organic phases, which separate polar metabolites into the aqueous layer and non-polar metabolites into the organic phase, which then can be easily separated for further analysis. While polar organic solvents such as methanol and acetonitrile can extract both hydrophobic and hydrophilic metabolites, non-polar solvents such as chloroform, MTBE and hexane are often used to only extract non-polar metabolites. During our method development stage, several different extraction solvent systems using these polar and non-polar solvents were tested. The polar organic solvents methanol or acetonitrile performed equally well for extracting metabolites, while the non-polar solvents chloroform, MTBE and hexane led to detectable differences in the total number of detected lipids. However, hexane was found to be the best performing non-polar solvent based on the data from SRM 1575a (data not shown) and was therefore used in combination with methanol to further optimize metabolite extraction by altering the sample-to-solvent ratios.

We extracted different amounts (mg) of the SRM pine needles with methanol and hexane to determine if more metabolites could be extracted with increasing amounts of starting material. Metabolites were extracted from 12.5 mg, 25 mg, 40 mg, and 50 mg of SRM pine needles using the same protocol described for 25 mg samples (Section 3.4). Twelve metabolites were chosen to represent the different metabolite classes and each of these were quantified and compared as a ratio to those obtained from 25 mg of sample. The ratios of these representative metabolites extracted from 12.5 mg-, 40 mg- and 50 mg-aliquots compared to 25 mg-aliquots are shown in Table 1. If no saturation occurred, we expect the theoretical ratios of the metabolites from 12.5 mg, 40 mg and 50 mg extracts to be 0.5, 1.6 and 2.0, respectively, relative to 25 mg. All the representative metabolites extracted from 12.5 mg produced the expected result, or 0.5 of the 25 mg aliquots. However, only the first six metabolites listed in Table 1 were extracted at the expected 1.6 and 2 ratios from 40 and 50 mg of starting material. The remaining six metabolites had less than the expected quantities extracted from 40 and 50 mg, suggesting that saturation of these metabolites was occurring. Thus, compared to using 25 mg of starting material, using 40 and 50 mg of starting material did not improve yields for all metabolites. While our results suggested that halving the starting material could yield similar ratios of metabolites to the 25 mg extract, some lipids detected in the 12.5 mg-aliquots were close to their LODs. Therefore, we chose 25 mg as the stating material amount for further extraction and validation of the assay.

### 2.2. Liquid Chromatography

**Figure 1:**
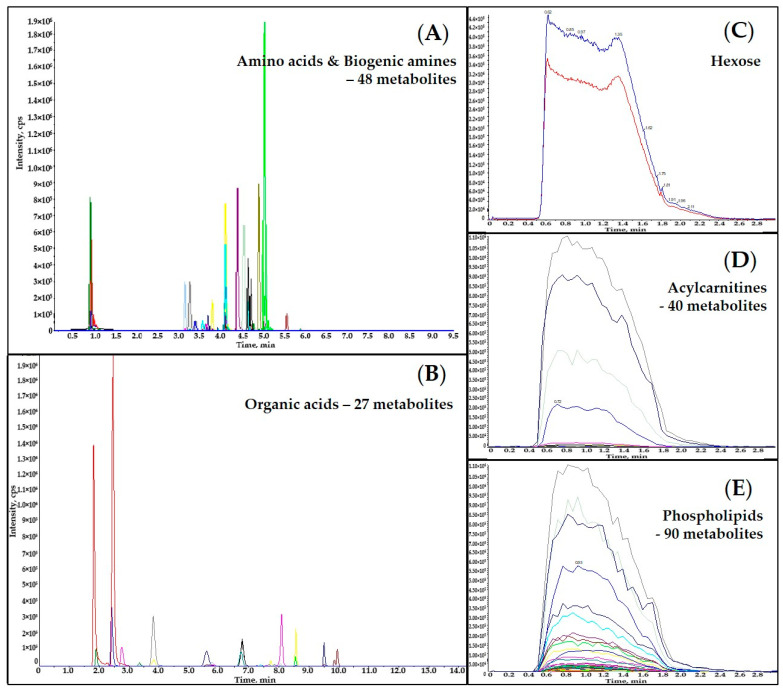
Overlaid representative Extracted Ion Chromatograms (EICs) of analyzed metabolites and ISTDs in an extracted pine needle sample: (**A**) EICs of amino acids, biogenic amines and their derivatives; (**B**) EICs of organic acids; (**C**) EICs of hexose; (**D**) EICs of acylcarnitines; (**E**) EICs of phospholipids.

Figure 1 displays overlaid representative extracted ion chromatograms (EICs) of an extracted pine needle sample. The LC-MS instrument analysis time for each sample was 9.5 min for amino acids and biogenic amines, 14 min for organic acids, 3 min for acylcarnitines, 3 min for phospholipids, and 3 min for hexose. Coelutions of certain pairs of metabolites were observed in both LC-MS/MS analyses (e.g., succinic acid and methylmalonic acid, leucine and isoleucine). However, all the co-eluted metabolites were separated by their distinct Q3 fragments (Table S2). All the metabolites and selected ISTDs analyzed using DFI-MS/MS (the acylcarnitines, hexose and phospholipids) were co-eluted and distinguished by their specific MRM transitions (Table S2).

### 2.3. Assay Validation

All the calibrants with concentrations provided in Table S1 were prepared in water or 75% aqueous methanol (as indicated) and stored in a −86 °C freezer until further analysis. The quantification of both LC-MS/MS analyses was performed based on the peak area ratios of the targeted analyte compared to corresponding isotope-labeled ISTD. A calibration regression curve was built for each analyte within its concentration range as described in Table S1. This wide range of concentrations was selected to cover those values that may be expected in different plant or food matrices. These plant concentrations were obtained from data available in online databases, such as FooDB [45]. The LOD, LOQ and regression parameters for all the analytes are summarized in Table S3, with selected representatives listed in Table 2 below. No significant carry-over was observed by comparing between the Cal 7 injection and the following double blank injection. Evaluations of intra-day and inter-day accuracy and precision were performed by analyzing QC samples at three different concentration levels (low, medium aTable nd high) on three different days. The results are listed in Table S4. Table 3 shows the accuracy and precision results of selected compounds among the 206 analyzed metabolites in this assay. With a formally defined criterion of the required accuracy being within 100 ± 20%, and precision within 20%, our results indicate that both LC-MS/MS analyses for this plant assay were sufficiently accurate and reproducible to meet our acceptance criteria. The recoveries of absolutely quantified metabolites at low, medium and high concentration levels in NIST^®^ SRM^®^ 1575a pine needle extracts (prepared according to Section 3.4) were also measured by comparing the calculated spiked concentration with the fortified amount. A summary of the recovery performance for all analytes is provided in Table S5, while a selected subset is listed in Table 4 as an example.

Due to the lack of individual authentic standards for each of those metabolites analyzed via the DFI-MS/MS method, namely acylcarnitines, hexose and phospholipids, only semi-quantification was done. LODs and LOQs were evaluated by the blank determination method mentioned above. Reproducibility was assessed by analyzing the above NIST^®^ SRM^®^ 1575a pine needle extracts in triplicate. Accuracy, precision and recoveries were calculated only for those analytes where authentic standards were available. All the results are summarized in Table S6, with some representative metabolites as examples summarized in Table 5.

### 2.4. Assay Application to Crop Plant Samples

We have already applied this targeted plant metabolomics assay to the analysis of a number of crop plants or plants of agricultural importance. This includes flower buds from six different cannabis strains, a large collection of canola root samples, as well as hundreds of different spruce and pine needle samples (pine and spruce trees are used to produce spruce and pine nuts in Canada). Table 6 summarizes the number of metabolites detected and quantified in different plant samples (all quantified metabolite concentrations for these samples are provided in Table S7). As can be seen from Table 6, the overall performance of this targeted LC-MS plant assay among different sample types is quite comparable. The one exception is the low number of phospholipids detected in cannabis buds, which may be due to the fact that flower buds are generally low in lipids. It may also be due to the fact that only dried, processed commercial cannabis samples were available for analysis as opposed to fresh samples. It is worth noticing that even though 40 acylcarnitines were screened in this assay, only free carnitine was consistently detected in all the samples. This is primarily because of the very low acylcarnitine content in most plant tissues sampled (which tend to be low in lipid or fatty acid content). However, we note that a number of long-chain acylcarnitines were detected in canola root samples. In addition to these primary metabolites, we were also able to detect and quantify most of the key plant hormones, including zeatin, jasmonic acid, abscisic acid, and salicylic acid.

While this assay is designed to detect 206 plant metabolites, we found that the average number of metabolites detected and quantified in any given plant sample was generally <110 compounds, or about 50% of the assay maximum. This is not unexpected. Indeed, this kind of partial coverage for other targeted LC-MS assays across different mammals and across different mammalian tissues or biofluids is also seen [46]. If we include all four plant species and all three plant tissues in our totals, we find that we were able to detect and quantify 143 different compounds. This suggests that if we characterized an even more diverse or metabolically active set of plant tissues (especially fruits, vegetables and nuts) or more diverse plant species, nearly full coverage (i.e., all 206 compounds) could be detected and quantified. The low number of detected acylcarnitines and the relatively high number of other expected compounds that were below the detection limit suggest that further refinements in the extraction protocol or adjustments to the sample volume requirements may be needed for certain plant tissue types. We also believe that the addition of other types of plant metabolites (especially more sugars or sugar alcohols as well as more polyphenols and common plant alkaloids) to the assay could make it even more useful. As the assay is easily extensible, efforts are underway in our laboratory to add these compounds to create a second, more expanded version of this plant assay.

While the main focus of this paper has been on this assay’s compound coverage and precision/accuracy, it is important to note that considerable effort also went into making this assay as efficient and as high-throughput as possible. In particular, by adapting the assay to a 96-well plate format and developing other high-throughput in-well extraction and separation procedures, we were able to create a workflow that allows us to consistently process 82 plant samples in as little as 45 h (about one sample every 30 min). This high-throughput capability allowed us to analyze more than 1500 spruce and pine needle samples on a single MS instrument.

## 3. Materials and Methods

### 3.1. Plant Material

To rigorously assess the performance of the assay, we decided to evaluate it on a number of diverse crop plant samples, including roots, flowers/buds and leaves from both annuals and perennials. Canola (*Brassica. napus* L. cv Westar) root samples as well as white spruce and lodgepole pine needle samples were obtained from our collaborators from local tree farms near the Edmonton region. For the spruce and pine needle samples, seedlings were grown in a greenhouse (18:6 h light:dark photoperiod, 60% relative humidity at 24 °C) in the summer for one growing season, lifted and placed into cold storage. The following spring, seedlings were repotted and allowed to grow in the greenhouse for nearly one month prior to sample collection and analysis. The six different cannabis strains (Alien Dawg, Tangerine Dream, Sensi Star, Quadra, Gabriola, and Island Honey) were purchased from a local cannabis store in Edmonton, Canada and stored at room temperature until further use. Cannabis has recently been legalized in Canada and it has become a significant agriculture product grown in greenhouses across Canada. The dried cannabis samples were stored at room temperature in a secure safe, while the canola root samples as well as spruce and pine needle samples were stored at −20 °C before they were analyzed.

### 3.2. Chemicals, Reagents and Materials

Optima™ LC/MS grade formic acid, Optima™ LC/MS grade methanol, acetonitrile and water, and Optima™ LC/MS grade ammonium acetate were purchased from Fisher Scientific (Ottawa, CA, USA). HPLC grade pyridine, chloroform, methyl tert-butyl ether, hexane and ethanol, phenylisothiocyanate (PITC), 3-nitrophenylhydrazines (3-NPH), 1-ethyl-3-(3-dimethylaminopropyl) carbodiimide (EDC), and butylated hydroxytoluene (BHT) were purchased from Sigma-Aldrich (Oakville, CA, USA). Chemical standards and corresponding isotope-labeled internal standards were purchased from C/D/N Isotopes Inc (Pointe-Claire, CA, USA), Cayman Chemical (Ann Arbor, MI, USA), Toronto Research Chemicals (North York, NY, USA), Cambridge Isotope Laboratories Inc. (Tewksbury, MA, USA), Sigma-Aldrich (Oakville, CA, USA), and IsoSciences (Ambler, PA, USA), except 3-(3-hydroxyphenyl)-3-hydroxypropionic acid (HPHPA). This compound and the ^13^C-labeled version of HPHPA were synthesized in-house [47]. Detailed information about the compounds and compound sourcing is provided in the Supplementary Document S1. NIST^®^ SRM^®^ 1575a pine needle material was purchased from Sigma-Aldrich (Oakville, CA, USA). Nunc^®^ 96 DeepWell™ plates and Multiscreen “solvinert” filter plates (hydrophobic, PTFE, 0.45 μm, clear, non-sterile) were both bought from Sigma-Aldrich (Oakville, CA, USA).

### 3.3. Stock Solutions, Internal Standard (ISTD) Mixtures, and Calibration Curve Standards

All chemicals used in this study were weighed individually on a Sartorius CPA225D micro-electronic balance (Mississauga, CA, USA) with a precision of 0.0001 g. Stock solutions, with specific concentrations for each analyte, were prepared by dissolving the accurately weighed chemicals in the corresponding solvents as indicated in Supplementary Table S1. Calibration curve standards, from calibration standard #1 (Cal 1) to calibration standard #7 (Cal 7)-detailed concentration levels, are also provided in Supplementary Table S1 and were prepared by mixing and diluting the corresponding stock solutions with solvents, covering different concentration ranges for different analytes according to their expected concentrations in different plants and plant tissues. Stock solutions of isotope-labeled standards (ISTDs) and the final working ISTD mixture solutions were also prepared the same way as described above. All calibration curve standards and ISTD working solutions were aliquoted and stored in a Thermo Scientific™ −86 °C freezer (Waltham, MA, USA) prior to further use.

### 3.4. Plant Extraction

For each plant sample, 25 mg of freeze-dried and homogenized plant sample was carefully weighed into a 1.5-mL Eppendorf tube and the measured exact mass was recorded for final calculation of metabolite concentrations per gram of dry plant weight. A total of 1 mL of a pre-cooled mixture of hexane and methanol (3:1, *v/v*) was added to the tube and vortexed thoroughly for 1 min, and then shaken at 1000 rpm on a Fisherbrand™ analog multitube vortexer (Ottawa, CA, USA) for 10 min at 4 °C. After shaking, the tube was sonicated in an ice-bath for another 10 min, followed by the addition of 500 µL of 25% aqueous methanol. The tube was again vortexed for 1 min to ensure complete mixing and centrifuged at 10,000 RCF (relative centrifugal force or × *g*) for 10 min at 4 °C. Two different layers (hexane layer and aqueous layer) were obtained after centrifugation and aliquoted into two separate clean Eppendorf tubes and used for further analysis.

### 3.5. Plant Extract Analysis

Two separate sample preparations involving two different pre-column derivatization reactions were developed for this targeted assay: (1) PITC derivatization targeting primary and secondary amine-containing metabolites, and (2) 3-NPH derivatization targeting keto- and carboxyl-containing metabolites. The PITC derivatization sample preparation method was used to obtain the concentrations of several classes of plant metabolites including amino acids and derivatives, biogenic amines and derivatives, hexose, and acylcarnitines. For this assay, 20 μL of the ISTD mixture solution and 10 μL of various standard solutions or sample extracts (50 percent aqueous methanol as a single blank sample, seven calibration standards, three different concentrations of quality control solutions, and plant extracts from aqueous layers) were pipetted directly onto the center of each corresponding spot in a 96-well multiscreen “solvinert” filter plate. After drying the plate under a gentle stream of nitrogen for 30 min, 50 μL of 5% PITC derivatization solution (where 300 μL of PITC reagent was added to a mixture containing 1900 μL each of ethanol, water and pyridine) was added to each well. The reaction was kept at room temperature for 20 min under quiescent conditions, followed by another 1-h drying period under a mild nitrogen stream to remove the excess PITC solution. To extract the targeted analytes, 300 μL of extraction solution (5 mM ammonium acetate in methanol) was then added to each well. The whole plate was covered and shaken on a rotary shaker at 450 rpm for 30 min at room temperature, and then centrifuged at 50 RCF for 5 min to collect the extracts from the 96-well filter plate to a Nunc^®^ 96 DeepWell™ plate. Finally, 150 μL of extracts was diluted with 150 μL water for LC-MS/MS analysis to absolutely quantify the amino acids, amino acid derivatives, the biogenic amines and their derivatives. The remaining extracts were diluted for direct flow injection-tandem mass spectrometry (DFI-MS/MS) analysis with 400 μL of DFI buffer (which consisted of 60 μL of formic acid, 10 mL of water and 290 mL of methanol) to quantify the acylcarnitines and hexose.

To quantify the phospholipids via DFI-MS/MS analysis, 10 μL of ISTD mixture solution and 5 μL of samples (50 percent aqueous methanol as a single blank sample, three quality control solutions, and plant extracts from hexane layers) were pipetted directly into the center of each well in a separate Nunc^®^ 96 DeepWell™ plate, followed by the addition of 150 μL of the same extraction solution as above and 600 μL of DFI buffer.

The 3-NPH derivatization method was used for the absolute quantification of organic acids. A total of 10 μL of the ISTD mixture solution and 10 μL of the samples (50 percent aqueous methanol as a single blank sample, seven calibration standards, three different concentrations of quality control solutions, and plant extracts from aqueous layers) were pipetted directly into the center of each individual well in a Nunc^®^ 96 DeepWell™ plate, followed by the addition of 30 μL of 75 percent aqueous methanol. A total of 25 μL each of the following three solutions: 3-NPH (250 mM prepared in 50 percent aqueous methanol), EDC (150 mM prepared in methanol) and pyridine (7.5% prepared in 75 percent aqueous methanol), was then added sequentially to each well of the plate. The whole plate was then shaken at 450 rpm on a rotary shaker for 2 h at room temperature to complete the derivatization reaction. After the reaction, 350 μL of water and 25 μL of BHT solution (2 mg/mL in methanol) were added to each well to dilute and stabilize the solution for LC-MS/MS analysis.

### 3.6. LC/DFI-MS/MS Analysis

An Agilent 1260 series UHPLC system (Palo Alto, CA, USA) coupled with an AB Sciex QTRAP^®^ 4000 mass spectrometer (Concord, ON, USA) was used for all online LC/DFI-MS/MS analyses. An Agilent reversed-phase Zorbax Eclipse XDB C18 column (3.0 mm × 100 mm, 3.5 μm particle size, 80 Å pore size) attached to a Phenomenex (Torrance, CA, USA) SecurityGuard C18 pre-column (4.0 mm × 3.0 mm) was used for the analysis of amino acids, biogenic amines, and organic acids. Red PEEK tubing was used to connect the LC system with the MS system for the analysis of lipids. The controlling software for the sample analysis was Analyst^®^ 1.5.3. The data analysis software was Analyst ^®^ 1.6.3. and MultiQuant 3.0.3.

The UHPLC parameters used to separate amino acids and biogenic amines were as follows: solvent A was 0.2% (*v/v*) formic acid (FA) in water, and solvent B was 0.2% (*v/v*) FA in acetonitrile. The gradient profile for this UHPLC elution was as follows: t = 0 min, 100% A; t = 0.5 min, 100% A; t = 5.5 min, 5% A; t = 6.5 min, 5% A; t = 7.0 min, 100% A; and maintained at 100% A for another 2.5 min until the column pressure was stable for the next injection. The flow rate was set to 0.5 mL/min, and 10 μL of the final sample solution was injected for analysis. The column oven was set at 50 °C. The QTRAP^®^ 4000 mass spectrometer was set to a positive electrospray ionization (ESI) mode with a scheduled multiple reaction monitoring (sMRM) scan. The IonSpray voltage was set at 5500 volts and the ion source temperature was set at 500 °C. The curtain gas (CUR), ion source gas 1 (GAS1), ion source gas 2 (GAS2) and collision gas (CAD) were set at 20, 40, 50 and medium, separately. The entrance potential (EP) was set to 15 volts, whereas the declustering potential (DP), collision energy (CE), collision cell exit potential (CXP), MRM precursor ion (Q1) and fragment ion (Q3) were optimized and set specifically for each analyte and isotope-labeled standard (ISTD).

For DFI-MS/MS analysis, the UHPLC autosampler was connected directly to the QTRAP^®^ 4000 MS Turbo V™ ion source via red PEEK tubing. The same DFI buffer used for sample dilution was again used as the mobile phase. The flow rate was set as follows: t = 0 min, 0.03 mL/min; t = 1.6 min, 0.03 mL/min; t = 2.4 min; 0.2 mL/min; t = 2.8 min, 0.2 mL/min; and t = 3.0 min, 0.03 mL/min. At the start of each run, 20 μL of each sample solution was injected into the QTRAP^®^ 4000 MS for analysis. The QTRAP^®^ 4000 MS was set to a positive ESI mode with MRM scanning to analyze phospholipids and acylcarnitines, and to a negative ESI mode to detect C-6 sugars. For the positive mode, the IonSpray voltage was set at 5500 volts and ion source temperature was set at 200 °C. The CUR, GAS1, GAS2 and CAD were set at 20, 40, 50 and medium, respectively. The EP and CXP were set at 10 volts and 15 volts, separately. For the negative mode, the IonSpray voltage was set at −4500 volts and the ion source temperature at 200 °C. The EP and CXP were set at −10 volts and −15 volts, respectively, whereas CUR, GAS1, GAS2 and CAD were set the same as positive mode. Likewise, the DP, CE, Q1 and Q3 were optimized and set individually for each analyte and ISTD.

To analyze the organic acids by LC-MS/MS, the elution solvents used were A) 0.01% (*v/v*) FA in water, and B) 0.01% (*v/v*) FA in acetonitrile. The elution gradient was set as follows: t = 0 min, 70% A; t = 1.5 min, 70% A; t = 8.0 min, 5% A; t = 8.01 min, 0% A; t = 9.5 min, 0% A; t = 9.6 min, 70% A; and t = 14.0 min, 70% A. The column oven was set at 40 °C. The flow rate was set at 0.3 mL/min, and the sample injection volume was 10 μL. The QTRAP^®^ 4000 mass spectrometer was set to a negative ESI mode with sMRM scanning. The IonSpray voltage was set at −4500 volts and the ion source temperature was set at 400 °C. The CUR, GAS1, GAS2 and CAD were set at 20, 30, 30 and medium, respectively. The EP was set at −10 volts, and the DP, CE, CXP, Q1 and Q3 were optimized and set individually for all the analytes and all ISTDs.

**Figure 2:**
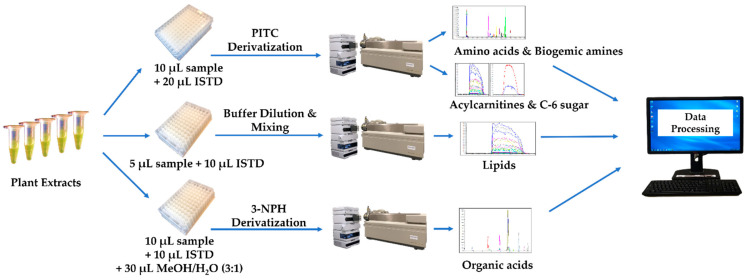
Schematic description of the plant extract analysis process.

All individually optimized MRM parameters including Q1, Q3, DP, CE, and CXP for each analyte are shown in Supplementary Table S2, and a scheme describing the whole process is shown in Figure 2.

### 3.7. Method Validation

#### 3.7.1. Calibration Regression

Calibration regression of the LC-MS/MS analysis assays for the quantification of amino acids, biogenic amines as well as organic acids was evaluated using seven standard mixture solutions prepared in the indicated solvents (water or 75% aqueous methanol) to create a seven-point calibration curve (Table S1). The ratios of each analyte’s peak area to its corresponding isotope-labeled ISTD’s peak area were plotted against the specifically prepared concentrations to build each analyte’s calibration curve separately, and the coefficient of determination (R^2^) was calculated and evaluated. Given the wide range of metabolite concentrations (i.e., up to an 80-fold variation between the lowest and highest concentration levels of each calibrant), quadratic regression with a 1/x weighting was used.

Due to the lack of individual chemical standards for each phospholipid and acylcarnitine, we analyzed these compounds via DFI-MS/MS in a semi-quantitative manner. In these cases, a single point calibration curve of a representative analyte was built so that it could be used for a group of analytes that share the same core structure, assuming linear regression through zero.

#### 3.7.2. Accuracy and Precision

To evaluate the accuracy and precision of all the analytes that were quantified by our LC-MS/MS methods, three different concentration levels (low, medium and high) were prepared in triplicate for each analyte and processed using the method described above. The measured concentrations were used to calculate the coefficient of variation (CV) expressed as a percentage.

#### 3.7.3. Recovery

Recovery was evaluated by analyzing several types of plant samples. Here, we will describe the recovery for pine needle/leaf extracts along with the extracts that had been spiked at three different concentrations (low, medium and high). All the spiked plant extracts as well as the non-spiked ones were prepared in triplicate. The recovery was calculated according to Equation (1), as shown below (where C_SE_ is the quantified concentration in the spiked extracts, C_NE_ is the quantified concentration in the non-spiked extracts, and C_A_ is the actual concentration of the analyte that was spiked), and expressed as a percentage.

(1) $$\mathrm{Recovery}\;\left(\%\right)=(C_{\mathrm{SE}}-{\;C}_{\mathrm{NE}})/C_{A}\times 100$$

#### 3.7.4. Limits of Detection (LOD) and Quantification (LOQ)

The LOD and LOQ were calculated according to the blank sample determination method, i.e., by analyzing seven replicates of the blank samples where a non-zero standard deviation was obtained, according to Equations (2) and (3) below (where C_Blank_ is the average concentration of the analyte in all blank samples, and SD_Blank_ is the standard deviation of the analyte concentrations measured in blank). However, for any analyte with a calculated standard deviation of zero, its LOD and LOQ were estimated using a signal-to-noise (*S/N*) method, where the analyte peak signal-to-noise ratio at a known concentration level was measured (via Analyst^®^ 1.6.3) and the concentrations of the analyte that would yield a *S/N* ratio equal to 3 or 10 would then be estimated to be LOD or LOQ.

(2) $$\mathrm{LOD}=C_{\mathrm{Blank}}+3\times {\mathrm{SD}}_{\mathrm{Blank}}$$

(3) $$\mathrm{LOQ}=C_{\mathrm{Blank}}+10\times {\mathrm{SD}}_{\mathrm{Blank}}$$

## 4. Conclusions

We have reported a comprehensive, high-throughput, targeted plant metabolomics assay for the quantification of up to 206 plant metabolites. In total, it measures 28 amino acids and derivatives, 27 organic acids, 20 biogenic amines and derivatives, 40 acylcarnitines, 90 phospholipids and C-6 sugars. This assay requires a minimal sample amount (~25 mg) while providing useful information about a wide range of primary and secondary metabolites. We have already applied this targeted plant metabolomics assay to the analysis of six different cannabis strains, dozens of canola root samples, and hundreds of spruce and pine needle samples. As the assays have been designed with efficiency and high-throughput sample processing in mind (using a 96-well plate format), we have found that it is possible to process up to 82 plant samples in as little as 45 h. As noted earlier, this assay has been extensively validated and tested. To date, more than 1500 crop plant samples from multiple plant species and multiple plant tissues have been analyzed using this assay. In addition, several manuscripts detailing the interesting and unexpected findings from these studies are now under development. As only minor modifications for the preparation of the different plant samples were required for our LC-MS plant assay, we are confident that this assay can be extended to analyze other types of plant and food samples (especially fruits, nuts and vegetables). Overall, we believe the development of a high-throughput, comprehensive targeted plant-specific metabolomic assay, such as the one described here, could play a significant role in improving the accuracy of characterizing, phenotyping and chemotyping many plants of agricultural and medicinal importance.

In addition to the assay described here, we have also created and are continuing to update several plant-specific or food-specific metabolome databases such as the Cannabis Compound Database (https://cannabisdatabase.ca/, accessed on 9 May 2021) and FooDB (https://foodb.ca/, accessed on 9 May 2021). These online metabolomic databases have been partially annotated through the plant assay described here. Overall, these web-accessible databases are intended to provide useful, quantitative information about the metabolites, metabolite concentrations and metabolic pathways of different crop plants and different plant food species. By combining both our LC-MS plant assay and these comprehensive metabolomic databases, we believe that it will be possible to develop a much more holistic understanding of the biochemistry and metabolism of many important crop plants.

## Figures and Tables

**Table 1:** Comparison of Ratios of Selected Metabolites Extracted from Three Different Quantities of Pine Needle SRM to a 25 mg Extract.

Metabolite	Concentration Comparison Ratios to 25 mg-Aliquots		
	12.5 mg-Aliquots	40 mg-Aliquots	50 mg-Aliquots
Succinic acid	0.495	1.66	1.89
Glutamine	0.517	1.62	1.97
Choline	0.517	1.61	2.01
Carnitine (C0)	0.509	1.58	1.96
Hexose	0.498	1.60	1.91
PC aa C38:6	0.494	1.58	2.06
Shikimic acid	0.516	1.29	1.33
Glyceric acid	0.517	1.28	1.31
Malic acid	0.509	1.27	1.44
Alanine	0.491	1.46	1.76
Arginine	0.511	1.48	1.65
LysoPC a C14:0	0.505	1.44	1.71

**Table 2:** Calibration Regression, LOD, LLOQ and ULOQ of Representative Analytes.

Analyte	Correlation Coefficient (R^2^)	LOD (μM)	LLOQ (μM)	ULOQ (μM)
Glycine	0.9990	0.859	25.0	2000
Proline	0.9994	0.136	10.0	800
Spermidine	0.9998	0.00954	0.250	20.0
Choline	0.9992	0.294	2.50	200
Shikimic acid	0.9992	0.321	1.25	100
Malic acid	0.9998	0.0574	1.25	100

**Table 3:** Intra- and Inter-day Accuracy and Precision of Representative Analytes.

Analyte		Intra-Day		Inter-Day	
	Fortified Concentration (μM)	Accuracy (%)	CV (%)	Accuracy (%)	CV (%)
Glycine	125	105	2.52	98.0	4.23
	500	95.7	2.43	103	1.33
	1500	96.3	3.90	97.5	3.25
Proline	50	112	1.63	96.7	4.36
	200	98.6	2.69	101	0.148
	600	96.9	2.07	101	2.44
Spermidine	1.25	103	2.29	107	8.30
	5.00	101	2.31	98.9	3.79
	15.0	98.1	3.37	95.9	5.15
Choline	12.5	111	2.03	101	2.57
	50.0	100	2.58	101	0.794
	150	103	2.42	101	1.95
Shikimic acid	2.50	95.7	1.68	97.0	2.09
	10.0	96.0	0.833	92.0	2.30
	50.0	101	2.65	98.4	0.704
Malic acid	2.50	97.0	3.23	99.9	1.33
	10.0	102	2.08	103	0.900
	50.0	104	0.223	102	2.67

**Table 4:** Recovery Performance of Representative Analytes in Spiked NIST^®^ SRM^®^ 1575a Pine Needle Extracts.

Analyte	Spiked Concentrations (μM)	Calculated Concentration (μM)	Recovery (%)
Glycine	125	129	103
	500	530	106
	1500	1646	110
Proline	50	53.3	107
	200	224	112
	600	616	103
Spermidine	1.25	1.39	111
	5.00	5.58	112
	15.0	17.1	114
Choline	12.5	12.5	100
	50.0	56.8	114
	150	158	105
Shikimic acid	2.50	2.58	103
	10.0	10.3	103
	50.0	46.0	91.9
Malic acid	2.50	2.83	113
	10.0	10.1	101
	50.0	53.9	108

**Table 5:** Validation Performance of Representative Analytes Analyzed via DFI-MS/MS.

Analyte	Accuracy (%)	CV (%)	Recovery (%)	LOD (μM)
LysoPC a C18:0	105	5.17	104	0.274
PC aa C36:0	104	6.22	101	0.0898
C0	92.6	4.28	98.5	0.222
Hexose	96.3	2.91	103	18.5

**Table 6:** Summary of Metabolite Numbers Detected and Quantified in Different Plant Types.

Metabolite Class	Number of Metabolites Detected and Quantified			
	NIST^®^ SRM^®^ 1575a Pine Needles	Canola Root Samples	Commercial Cannabis Buds	Spruce and Pine Needles
Amino acids and derivatives	24	27	27	26
Biogenic amines and derivatives	8	12	13	14
Organic acids (phytohormones included)	15	21	24	18
Acylcarnitines	1	6	1	1
Phospholipids	44	43	12	46
Hexose	1	1	1	1
Total	93	110	78	106

## Data Availability

The data presented in this study are available in article.

## Supplementary Materials

The following are available online at https://www.mdpi.com/article/10.3390/metabo11050303/s1, Table S1: Concentration of Standards at Different Calibration Levels, Table S2: Optimized MRM Transitions and Mass Spectrometer Parameters, Table S3: Calibration Regression, LOD and LOQ, Table S4: Intra- and Inter-day Accuracy and Precision, Table S5: Recovery Performance in Spiked NIST® SRM® 1575a Pine Needle Extracts, Table S6: Validation Performance of Analytes Analyzed via DFI-MS/MS, Table S7: Detected and Quantified Plant Metabolite Concentrations in Different Samples. Supplementary Document S1: Detailed chemical vendor information.
