# Supplementary material for: A Comprehensive Targeted Metabolomics Assay for Crop Plant Sample Analysis
Source: Metabolites. 2021 May 11;11(5):303. doi: 10.3390/metabo11050303 (PMC8151637; doi:10.3390/metabo11050303)
Supplement: Supplementary file 1 [file metabolites-11-00303-s001.zip › Supplementary Document S1.pdf]

Supplementary Document S1.

Detailed chemical vendor information

Chemical standards including asymmetric dimethylarginine (ADMA), carnosine, creatinine, levodopa (DOPA), dopamine, histamine, trans-hydroxyproline, phenylethylamine, putrescine, sarcosine, serotonin, spermidine, spermine, taurine, tyramine, alanine, arginine, asparagine, aspartic acid, citrulline, glutamine, glutamic acid, glycine, histidine, leucine, isoleucine, lysine, methionine, ornithine, phenylalanine, proline, serine, threonine, tryptophan, valine, 3-methylhistidine, betaine, trimethylamine N-oxide (TMAO), choline, nitro-tyrosine, tyrosine, kynurenine, creatine, homoserine, zeatin, shikimic acid, glyceric acid, lactic acid, beta-hydroxybutyric acid, alpha-ketoglutaric acid, citric acid, butyric acid, propionic acid, succinic acid, fumaric acid, pyruvic acid, hippuric acid, methylmalonic acid, 5-hydroxyindoleacetic acid, para-hydroxyphenylacetic acid, malic acid, glutaric acid, valeric acid, benzoic acid, oxalic acid, oxaloacetic acid, salicylic acid, abscisic acid, trans-aconitic acid, jasmonic acid, indole-3-acetic acid, L-carnitine inner salt, acetyl-L-carnitine hydrochloride, propionyl-L-carnitine, butyryl-L-carnitine, hexanoyl-L-carnitine, octanoyl-L-carnitine, decanoyl-L-carnitine, dodecanoyl-L-carnitine, tetradecanoyl-L-carnitine, hexadecanoyl-L-carnitine, octadecanoyl-L-carnitine, N-stearoyl-D-erythro-sphingosylphosphorylcholine, 1,2-dilinolenoyl-sn-glycero-3-phosphocholine, 1,2-Dioctadecanoyl-sn-glycero-3-phosphocholine, 1-oleoyl-2-hydroxy-sn-glycero-3-phosphocholine, and glucose were all bought from Sigma-Aldrich (Oakville, CA). N_1, N_{12}-diacetylspermine hydrochloride and proline-betaine were purchased from Cayman Chemical (Ann Arbor, MI, U.S.A.). Other stable isotope (D, ^{13}C, ^{15}N)-labelled internal standards (ISTD) including D_2-ornithine, ^{15}N-histidine, D_3-creatinine, D_3-DOPA, D_4-dopamine, ^{13}C-tyrosine, ^{13}C-D_3-methionine, D_3-proline, D_4-serotonin, D_4-putrescine, D_3-sarcosine, $^{13}C_2$-taurine, D_4-tyramine, ^{15}N-alanine, $^{13}C_6$-arginine, ^{15}N-asparagine, D_3-aspartic acid, D_7-citrulline, D_3-glutamic acid, D_5-glutamine, $^{13}C_2$-glycine, ^{13}C-leucine, ^{15}N-phenylalanine, ^{13}C-serine, D_2-

threonine, $^{15}N_2$-tryptophan, D_8-valine, D_9-TMAO and $^{15}N_2$-uric acid were bought from Cambridge Isotope Laboratories Inc. (Tewksbury, MA, U.S.A.). D_8-spermine tetrahydrochloride, D_8-spermidine trihydrochloride were purchased from IsoSciences (Ambler, U.S.A.). D_6-N_1, N_{12}-diacetylspermine dihydrochloride and D_6-ADMA were bought from Toronto Research Chemicals (North York, CA). D_3-creatinine, D_9-choline chloride, D_9-betaine hydrochloride, 2-D_1-sodium L-lactate, D_4-sodium beta-hydroxybutyrate, 1-^{13}C-alpha-ketoglutaric acid, D_4-citric acid, 1-^{13}C-butyric acid, 1-^{13}C-propionic acid, D_4-succinic acid, $^{13}C_2$-D_2-fumaric acid, ^{13}C-pyruvic acid, D_2-hippuric acid, methyl-D_3-malonic acid, and D_2-indole-3-acetic acid were purchased from C/D/N Isotopes Inc. (Pointe-Claire, CA). Labelled carnitine standard set was purchased from Cambridge Isotope Laboratories Inc. (Tewksbury, U.S.A.). 1,2-dimyristoyl-sn-glycero-3-phosphocholine, 1,2-diarachidoyl-sn-glycero-3-phosphocholine, 1-nonanoyl-2-hydroxy-sn-glycero-3-phosphocholine, N-hexanoyl-D-erythro-sphingosylphosphorylcholine, and $^{13}C_6$-glucose were bought from Sigma-Aldrich (Oakville, CA).
