# Supplementary material for: A Comprehensive Targeted Metabolomics Assay for Crop Plant Sample Analysis
Source: Metabolites. 2021 May 11;11(5):303. doi: 10.3390/metabo11050303 (PMC8151637; doi:10.3390/metabo11050303)
Supplement: Supplementary file 1 [file metabolites-11-00303-s001.zip › Supplementary Table S1.pdf]

Supplementary Table S1. Concentration of Standards at Different C[illegible]						
Analyte	Concentration in μM					
	Cal1	Cal2	Cal3	Cal4	Cal5	Cal6
Creatinine	10	20	100	200	400	600
Glycine	25	50	250	500	1000	1500
Alanine	20	40	200	400	800	1200
Serine	5	10	50	100	200	300
Histamine	1	2	10	20	40	60
Proline	10	20	100	200	400	600
Valine	10	20	100	200	400	600
Threonine	5	10	50	100	200	300
Phenylethylamine	0.1	0.2	1	2	4	6
Taurine	2.5	5	25	50	100	150
Putrescine	0.1	0.2	1	2	4	6
trans-Hydroxyproline	1	2	10	20	40	60
Leucine	5	10	50	100	200	300
Isoleucine	5	10	50	100	200	300
Asparagine	5	10	50	100	200	300
Aspartic acid	5	10	50	100	200	300
Glutamine	20	40	200	400	800	1200
Glutamic acid	10	20	100	200	400	600
Methionine	5	10	50	100	200	300
Dopamine	1	2	10	20	40	60
Histidine	5	10	50	100	200	300
Phenylalanine	5	10	50	100	200	300
Arginine	5	10	50	100	200	300
Citrulline	5	10	50	100	200	300
Serotonin	0.1	0.2	1	2	4	6
Tyrosine	5	10	50	100	200	300
DOPA	0.5	1	5	10	20	30
Asymmetric dimethylarginine	0.25	0.5	2.5	5	10	15
Total dimethylarginine	0.25	0.5	2.5	5	10	15
Tryptophan	5	10	50	100	200	300
Kynurenine	1	2	10	20	40	60
Carnosine	0.5	1	5	10	20	30
Nitro-Tyrosine	1	2	10	20	40	60
Ornithine	5	10	50	100	200	300
Lysine	10	20	100	200	400	600
Spermidine	0.25	0.5	2.5	5	10	15
Spermine	0.25	0.5	2.5	5	10	15

Sarcosine	1	2	10	20	40	60
Diacetylspermine	0.25	0.5	2.5	5	10	15
Tyramine	0.5	1	5	10	20	30
Creatine	2.5	5	25	50	100	150
Betaine	5	10	50	100	200	300
Choline	2.5	5	25	50	100	150
Trimethylamine N-oxide	10	20	100	200	400	600
Methylhistidine	1	2	10	20	40	60
Proline-Betaine	20	40	200	400	800	1200
Zeatin	1.25	2.5	12.5	25	50	75
Homoserine	2.5	5	25	50	100	150
Shikimic acid	1.25	2.5	5	10	20	40
Glyceric acid	2.5	5	10	20	40	80
beta-Hydroxybutyric acid	5	10	20	40	80	160
Lactic acid	50	100	200	400	800	1600
HPHPA	0.1	0.2	0.4	0.8	1.6	3.2
Propionic acid	0.55	1.1	2.2	4.4	8.8	17.6
5-Hydroxyindoleacetic acid	0.02	0.04	0.08	0.16	0.32	0.64
para-Hydroxyphenylacetic aci	0.2	0.4	0.8	1.6	3.2	6.4
Malic acid	1.25	2.5	5	10	20	40
Butyric acid	0.2	0.4	0.8	1.6	3.2	6.4
Hippuric acid	0.5	1	2	4	8	16
Succinic acid	1	2	4	8	16	32
Glutaric acid	2.5	5	10	20	40	80
Methylmalonic acid	0.05	0.1	0.2	0.4	0.8	1.6
Fumaric acid	0.5	1	2	4	8	16
Valeric acid	0.2	0.4	0.8	1.6	3.2	6.4
Benzoic acid	0.5	1	2	4	8	16
Oxalic acid	1.25	2.5	5	10	20	40
Indole acetic acid	0.1	0.2	0.4	0.8	1.6	3.2
Oxaloacetic acid	1.25	2.5	5	10	20	40
Salicylic acid	1.25	2.5	5	10	20	40
Citric acid	5	10	20	40	80	160
Abscisic acid	0.125	0.25	0.5	1	2	4
Aconitic acid	0.125	0.25	0.5	1	2	4
Jasmonic acid	0.125	0.25	0.5	1	2	4
Pyruvic acid	2.5	5	10	20	40	80
alpha-Ketoglutaric acid	0.5	1	2	4	8	16

alibration Levels	
Cal7	Solvents
800	
2000	
1600	
400	
80	
800	
800	
400	
8	
200	
8	
80	
400	
400	
400	
400	
1600	
800	
400	
80	
400	
400	
400	
400	Water
8	
400	
40	
20	
20	
400	
80	
40	
80	
400	
800	
20	
20	

80	
20	
40	
200	
400	
200	
800	
80	
1600	
100	
200	
100	
200	
400	
4000	
8	
44	
1.6	
16	
100	
16	
40	
80	
200	
4	75% aqueous methanol
40	
16	
40	
100	
8	
100	
100	
400	
10	
10	
10	
200	
40	
