# Supplementary material for: A Comprehensive Targeted Metabolomics Assay for Crop Plant Sample Analysis
Source: Metabolites. 2021 May 11;11(5):303. doi: 10.3390/metabo11050303 (PMC8151637; doi:10.3390/metabo11050303)
Supplement: Supplementary file 1 [file metabolites-11-00303-s001.zip › Supplementary Table S2.pdf]

Supplementary Table S2. Optimized MRM Transitions and Mass Spectrometer Parameters					
Analyte	MRM Optimization				
	Q1	Q3	DP	CE	CXP
Creatinine	114.1	44.1	60	17	15
Glycine	211.2	75.9	66	14	15
Alanine	225.2	44.2	61	35	15
Serine	241.2	60	41	31	15
Histamine	247	154	56	19	15
Proline	251.2	70.3	51	43	15
Valine	253.2	72.2	81	31	15
Threonine	255.2	74.1	66	29	15
Phenylethylamine	257.2	105.2	71	31	15
Taurine	261	126.1	71	21	15
Putrescine	266.1	113.9	100	25	15
trans-Hydroxyproline	267.15	68	56	61	15
Leucine	267.3	43	55	66	15
Isoleucine	267.3	69	55	45	15
Asparagine	268.2	87	41	25	15
Aspartic acid	269.2	116.2	76	21	15
Glutamine	282.2	130	71	25	15
Glutamic acid	283.2	130.2	76	23	15
Methionine	285.1	104.2	71	25	15
Dopamine	289.2	137.2	56	29	15
Histidine	291.1	110.2	56	33	15
Phenylalanine	301.2	120.2	71	31	15
Arginine	310	217	76	23	15
Citrulline	311.2	113.1	56	29	15
Serotonin	312.3	160.2	66	27	15
Tyrosine	317.2	136.1	76	31	15
DOPA	333.1	198.1	61	19	15
Asymmetric dimethylarginine	338.2	46	71	61	15
Total dimethylarginine	338.2	70.1	71	61	15
Tryptophan	340.2	188.2	96	25	15
Kynurenine	344.2	146.2	46	33	15
Carnosine	362.2	110.1	46	47	15
Nitro-Tyrosine	362.2	136.1	65	41	15
Ornithine	403.2	310.2	76	19	15
Lysine	417.2	324.2	81	19	15
Spermidine	551.2	193.2	71	41	15
Spermine	743.3	193.2	56	61	15

Sarcosine	225.1	44.2	43	31	15
Diacetylspermine	422.4	171.2	32	34	15
Tyramine	273.2	121.1	41	29	15
Creatine	132.1	90	40	18	15
Betaine	118.1	59.2	60	30	15
Choline	104.2	60.1	28	50	15
Trimethylamine N-oxide	76	58	40	26	15
Methylhistidine	305.1	212.1	46	23	15
Proline-Betaine	114.1	84	41	31	15
Zeatin	220.1	136.1	71	27	10
Homoserine	255.1	120.1	51	19	8
Shikimic acid	308	290	-67	-20	-6
Glyceric acid	239.9	136.9	-60	-25	-9
beta-Hydroxybutyric acid	238	193.8	-58	-21	-6
Lactic acid	224	137	-55	-12	-10
HPHPA	316.2	194.2	-70	-20	-9
Propionic acid	208	136.8	-30	-26	-25
5-Hydroxyindoleacetic acid	325	132.1	-84	-30	-5
para-Hydroxyphenylacetic acid	286	150	-80	-27	-7
Malic acid	404.1	208	-80	-26	-13
Butyric acid	221.9	136.8	-68	-30	-5
Hippuric acid	313	136.9	-65	-26	-11
Succinic acid	387.1	234	-100	-27	-20
Glutaric acid	401.2	248	-85	-26	-5
Methylmalonic acid	387	177.9	-85	-28	-9
Fumaric acid	385	234	-78	-20	-9
Valeric acid	236	137.1	-75	-24	-5
Benzoic acid	256	250	-60	-26	-9
Oxalic acid	359.1	149.7	-65	-30	-7
Indole acetic acid	308.9	155.9	-100	-28	-16
Oxaloacetic acid	401.1	383.1	-60	-16	-9
Salicylic acid	272	117.9	-65	-46	-7
Citric acid	596.3	221.9	-92	-50	-16
Abscisic acid	398.1	244.1	-80	-24	-13
Aconitic acid	578.1	424.7	-75	-22	-11
Jasmonic acid	344.1	194.3	-70	-26	-4
Pyruvic acid	357	136.8	-80	-33	-5
alpha-Ketoglutaric acid	550.1	232.7	-104	-36	-24
Hexose	179	89	-55	-12	-15
C0	162.1	85.1	61	27	15
C2	204.1	85.1	41	27	15
C3:1	216.1	85.1	49	27	15
C3	218.1	85.1	46	29	15

C4:1	230.1	85.1	52	29	15
C4	232.2	85.1	46	29	15
C3OH	234.1	85.1	53	30	15
C5:1	244.2	85.1	55	31	15
C5	246.2	85.1	46	29	15
C4OH	248.1	85.1	55	32	15
C6:1	258.1	85.1	57	33	15
C6	260.2	85.1	56	27	15
C5OH	262.2	85.1	58	33	15
C5:1DC	274.1	85.1	60	35	15
C5DC	276.2	85.1	61	35	15
C8	288.2	85.1	66	33	15
C5MDC	290.2	85.1	63	37	15
C9	302.2	85.1	66	39	15
C7DC	304.2	85.1	66	39	15
C10:2	312.2	85.1	67	40	15
C10:1	314.2	85.1	68	40	15
C10	316.2	85.1	56	37	15
C12:1	342.2	85.1	73	44	15
C12	344.3	85.1	73	44	15
C14:2	368.3	85.1	78	47	15
C14:1	370.3	85.1	78	47	15
C14	372.2	85.1	86	45	15
C12DC	374.3	85.1	86	45	15
C14:2OH	384.3	85.1	81	49	15
C14:1OH	386.3	85.1	81	50	15
C16:2	396.3	85.1	83	51	15
C16:1	398.3	85.1	84	51	15
C16	400.3	85.1	84	51	15
C16:2OH	412.3	85.1	86	53	15
C16:1OH	414.3	85.1	87	53	15
C16OH	416.3	85.1	87	53	15
C18:2	424.3	85.1	89	54	15
C18:1	426.4	85.1	89	55	15
C18	428.4	85.1	96	63	15
C18:1OH	442.4	85.1	92	57	15
LysoPC a C14:0	468.3	184	79	30	15
LysoPC a C16:1	494.3	184	79	30	15
LysoPC a C16:0	496.3	184	81	31	15
LysoPC a C17:0	510.3	184	82	31	15
LysoPC a C18:2	520.3	184	82	31	15
LysoPC a C18:1	522.3	184	82	31	15
LysoPC a C18:0	524.3	184	85	31	15

LysoPC a C20:4	544.3	184	85	31	15
LysoPC a C20:3	546.3	184	87	31	15
LysoPC a C24:0	608.4	184	96	33	15
LysoPC a C26:1	634.4	184	98	33	15
LysoPC a C26:0	636.5	184	101	34	15
LysoPC a C28:1	662.5	184	125	53	15
LysoPC a C28:0	664.5	184	105	35	15
PC aa C24:0	622.4	184	98	33	15
PC aa C26:0	650.5	184	103	35	15
PC aa C28:1	676.5	184	106	35	15
PC ae C30:2	688.5	184	108	36	15
PC ae C30:1	690.5	184	109	36	15
PC ae C30:0	692.6	184	111	37	15
PC aa C30:2	702.5	184	111	37	15
PC aa C30:0	706.5	184	114	37	15
PC ae C32:2	716.6	184	114	37	15
PC ae C32:1	718.6	184	115	38	15
PC aa C32:3	728.5	184	117	38	15
PC aa C32:2	730.5	184	117	38	15
PC aa C32:1	732.6	184	118	39	15
PC aa C32:0	734.6	184	119	39	15
PC ae C34:3	742.6	184	120	39	15
PC ae C34:2	744.6	184	120	39	15
PC ae C34:1	746.6	184	121	39	15
PC ae C34:0	748.6	184	122	40	15
PC aa C34:4	754.5	184	122	40	15
PC aa C34:3	756.6	184	123	40	15
PC aa C34:2	758.6	184	123	40	15
PC aa C34:1	760.6	184	125	41	15
PC ae C36:5	766.6	184	125	41	15
PC ae C36:4	768.6	184	126	41	15
PC ae C36:3	770.6	184	126	41	15
PC ae C36:2	772.6	184	127	41	15
PC ae C36:1	774.6	184	127	41	15
PC ae C36:0	776.7	184	128	42	15
PC aa C36:6	778.5	184	128	42	15
PC aa C36:5	780.6	184	128	42	15
PC aa C36:4	782.6	184	129	42	15
PC aa C36:3	784.6	184	130	42	15
PC aa C36:2	786.6	184	130	42	15
PC aa C36:1	788.6	184	131	43	15
PC aa C36:0	790.6	184	131	43	15
PC ae C38:6	792.6	184	132	43	15

PC ae C38:5	794.6	184	132	43	15
PC ae C38:4	796.6	184	133	43	15
PC ae C38:3	798.6	184	133	43	15
PC ae C38:2	800.7	184	133	43	15
PC ae C38:1	802.7	184	134	44	15
PC ae C38:0	804.7	184	135	44	15
PC aa C38:6	806.6	184	135	44	15
PC aa C38:5	808.6	184	136	44	15
PC aa C38:4	810.6	184	136	44	15
PC aa C38:3	812.6	184	136	44	15
PC aa C38:1	816.7	184	138	45	15
PC aa C38:0	818.7	184	138	45	15
PC ae C40:6	820.6	184	139	45	15
PC ae C40:5	822.6	184	139	45	15
PC ae C40:4	824.7	184	140	45	15
PC ae C40:3	826.7	184	140	46	15
PC ae C40:2	828.7	184	141	46	15
PC ae C40:1	830.7	184	141	46	15
PC aa C40:6	834.6	184	143	46	15
PC aa C40:5	836.6	184	143	47	15
PC aa C40:4	838.6	184	144	47	15
PC aa C40:3	840.7	184	144	47	15
PC aa C40:2	842.7	184	144	47	15
PC aa C40:1	844.7	184	145	47	15
PC ae C42:5	850.7	184	147	48	15
PC ae C42:4	852.7	184	148	48	15
PC ae C42:3	854.7	184	148	48	15
PC ae C42:2	856.7	184	149	48	15
PC ae C42:1	858.7	184	141	46	15
PC ae C42:0	860.8	184	150	49	15
PC aa C42:6	862.6	184	150	49	15
PC aa C42:5	864.7	184	151	49	15
PC aa C42:4	866.7	184	152	50	15
PC aa C42:2	870.7	184	153	50	15
PC aa C42:1	872.7	184	153	50	15
PC aa C42:0	874.7	184	154	50	15
PC ae C44:6	876.7	184	154	50	15
PC ae C44:5	878.7	184	155	51	15
PC ae C44:4	880.7	184	156	51	15
PC ae C44:3	882.7	184	156	51	15
