# Supplementary material for: A Comprehensive Targeted Metabolomics Assay for Crop Plant Sample Analysis
Source: Metabolites. 2021 May 11;11(5):303. doi: 10.3390/metabo11050303 (PMC8151637; doi:10.3390/metabo11050303)
Supplement: Supplementary file 1 [file metabolites-11-00303-s001.zip › Supplementary Table S3.pdf]

Supplementary Table S3. Calibration Regression, LOD and LOQ

Analyte	Correlation Coefficient (R^2)	LOD (μM)
Creatinine	0.9988	0.443
Glycine	0.9990	0.859
Alanine	0.9970	0.341
Serine	0.9988	0.0424
Histamine	0.9968	0.0136
Proline	0.9994	0.136
Valine	0.9996	0.161
Threonine	0.9998	0.185
Phenylethylamine	0.9998	0.00640
Taurine	0.9996	0.0346
Putrescine	0.9998	0.00750
trans-Hydroxyproline	0.9990	0.0566
Leucine	0.9996	0.652
Isoleucine	0.9970	0.0525
Asparagine	0.9976	0.482
Aspartic acid	0.9980	0.968
Glutamine	0.9988	0.266
Glutamic acid	0.9994	0.255
Methionine	0.9976	0.0291
Dopamine	0.9994	0.0615
Histidine	0.9986	0.114
Phenylalanine	0.9974	0.0147
Arginine	0.9982	0.110
Citrulline	0.9992	0.750
Serotonin	0.9986	0.0158
Tyrosine	0.9966	0.0102
DOPA	0.9990	0.0719
Asymmetric dimethylarginine	0.9994	0.115
Total dimethylarginine	0.9998	0.00898
Tryptophan	0.9988	0.0599
Kynurenine	0.9954	0.0164
Carnosine	0.9988	0.0274
Nitro-Tyrosine	0.9974	0.0346
Ornithine	0.9994	0.289
Lysine	0.9980	0.155
Spermidine	0.9992	0.00954
Spermine	0.9998	0.0568
Sarcosine	0.9970	0.167
Diacetylspermine	0.9946	0.0974

Tyramine	0.9994	0.0336
Creatine	0.9998	0.180
Betaine	0.9986	1.23
Choline	0.9992	0.294
Trimethylamine N-oxide	0.9996	0.266
Methylhistidine	0.9986	0.00708
Proline-Betaine	0.9990	0.811
Zeatin	0.9950	0.543
Homoserine	0.9906	0.0223
Shikimic acid	0.9992	0.321
Glyceric acid	0.9998	0.0395
beta-Hydroxybutyric acid	0.9998	0.0763
Lactic acid	0.9994	0.872
HPHPA	0.9978	0.00877
Propionic acid	0.9994	0.0344
5-Hydroxyindoleacetic acid	0.9968	0.0757
para-Hydroxyphenylacetic acid	0.9978	0.0694
Malic acid	0.9998	0.0574
Butyric acid	0.9996	0.0777
Hippuric acid	0.9998	0.0211
Succinic acid	1.0000	0.0215
Glutaric acid	0.9998	0.0268
Methylmalonic acid	0.9986	0.0105
Fumaric acid	0.9996	0.0303
Valeric acid	0.9998	0.0549
Benzoic acid	0.9996	0.0141
Oxalic acid	0.9952	0.104
Indole acetic acid	0.9952	0.0171
Oxaloacetic acid	0.9978	0.0846
Salicylic acid	0.9992	0.0571
Citric acid	0.9998	0.177
Abscisic acid	0.9986	0.0394
Aconitic acid	0.9990	0.0291
Jasmonic acid	0.9996	0.0897
Pyruvic acid	0.9920	0.0875
alpha-Ketoglutaric acid	0.9960	0.0248

**LOQ (μM)**
1.48
2.86
1.13
0.141
0.0454
0.453
0.537
0.617
0.0213
0.115
0.0250
0.189
2.17
0.175
1.61
3.23
0.886
0.851
0.0969
0.205
0.378
0.0489
0.366
2.50
0.0526
0.0340
0.240
0.385
0.0299
0.200
0.0548
0.0914
0.115
0.963
0.516
0.0318
0.189
0.556
0.325

0.112
0.601
4.10
0.980
0.886
0.0236
2.70
1.81
0.0743
1.07
0.132
0.254
2.91
0.0292
0.115
0.252
0.231
0.191
0.259
0.0703
0.0717
0.0893
0.0350
0.101
0.183
0.0470
0.347
0.0570
0.282
0.190
0.590
0.131
0.0970
0.299
0.292
0.0827
