# Supplementary material for: A Comprehensive Targeted Metabolomics Assay for Crop Plant Sample Analysis
Source: Metabolites. 2021 May 11;11(5):303. doi: 10.3390/metabo11050303 (PMC8151637; doi:10.3390/metabo11050303)
Supplement: Supplementary file 1 [file metabolites-11-00303-s001.zip › Supplementary Table S4.pdf]

Supplementary Table S4. Intra- and Inter-day Accuracy and Precision

Analyte	Low				Medium			
	Intra-day		Inter-day		Intra-day		Inter-day	
	Accuracy (%)	CV (%)	Accuracy (%)	CV (%)	Accuracy (%)	CV (%)	Accuracy (%)	CV (%)
Creatinine	100	3.72	101	2.90	105	4.46	99.4	2.08
Glycine	105	2.52	98.0	4.23	95.7	2.43	103	1.33
Alanine	101	2.56	96.0	1.74	101	0.991	98.8	2.46
Serine	108	9.82	96.0	4.62	98.3	5.14	98.8	1.08
Histamine	110	3.46	104	1.53	97.7	7.07	104	2.77
Proline	112	1.63	96.7	4.36	98.6	2.69	101	0.148
Valine	112	4.96	96.7	2.41	102	2.54	100	1.83
Threonine	95.4	6.44	100	5.96	88.8	8.30	102	1.21
Phenylethylamine	98.4	4.27	105	7.87	108	2.03	101	5.01
Taurine	103	8.50	100	4.65	97.3	2.43	93.4	2.31
Putrescine	101	9.53	94.9	6.15	106	5.10	95.7	6.23
trans-Hydroxyproline	101	8.97	101	5.33	101	7.74	99.1	1.13
Leucine	103	4.74	99.7	2.50	91.5	3.16	98.6	1.70
Isoleucine	104	1.27	114	0.154	98.5	1.46	102	2.67
Asparagine	109	7.16	106	5.73	110	5.14	102	8.01
Aspartic acid	96.3	6.97	93.6	3.54	105	9.25	94.2	8.93
Glutamine	101	2.15	99.6	6.74	101	4.95	98.7	3.51
Glutamic acid	106	6.44	93.7	9.10	103	10.5	101	11.9
Methionine	107	3.63	96.3	4.85	104	3.32	99.4	1.14
Dopamine	98.9	0.193	100	5.50	96.1	3.59	101	0.721
Histidine	105	0.138	92.3	0.854	97.9	2.88	104	0.422
Phenylalanine	109	1.17	98.0	3.73	92.1	3.48	100	0.330
Arginine	107	4.19	96.3	4.44	104	2.63	99.5	1.02
Citrulline	102	6.11	96.6	4.14	102	6.25	99.4	2.11

Serotonin	91.0	6.53	102	0.857	98.0	5.38	98.0	4.31
Tyrosine	104	3.53	90.2	1.78	98.5	4.68	93.3	4.54
DOPA	106	2.79	99.1	6.25	96.9	5.28	93.2	2.18
Asymmetric dimethylarginine	94.0	6.13	101	1.65	105	3.23	92.0	1.28
Total dimethylarginine	102	1.48	91.6	8.49	101	6.39	98.8	2.72
Tryptophan	110	0.91	99.0	3.16	100	1.65	98.8	1.53
Kynurenine	100	4.34	87.7	6.42	98.5	6.83	94.9	1.92
Carnosine	99.0	3.16	108	6.35	97.1	5.00	99.0	2.25
Nitro-Tyrosine	103	9.49	106	6.62	99.2	1.01	107	5.88
Ornithine	111	3.04	97.1	3.71	97.5	5.13	98.7	2.46
Lysine	104	5.99	98.1	6.01	90.8	6.55	96.9	0.696
Spermidine	103	2.29	107	8.30	101	2.31	98.9	3.79
Spermine	106	6.06	111	8.00	99.5	4.73	96.9	8.76
Sarcosine	100	0.733	102	7.94	106	0.679	101	1.26
Diacetylspermine	102	4.29	110	6.66	108	7.94	98.2	3.27
Tyramine	98.7	4.45	98.1	9.34	100	3.43	101	1.61
Creatine	98.3	4.32	99.4	4.09	105	2.78	98.2	0.878
Betaine	96.8	2.88	90.3	10.3	105	5.54	101	0.239
Choline	111	2.03	101	2.57	100	2.58	101	0.794
Trimethylamine N-oxide	105	1.31	101	3.85	100	2.53	99.9	1.97
Methylhistidine	102	1.16	94.2	8.54	97.9	2.48	100	1.02
Proline-Betaine	108	3.79	107	4.14	105	3.19	93.9	3.21
Zeatin	110	4.09	108	2.09	98.4	3.95	107	6.42
Homoserine	97.8	4.83	99.9	5.22	90.1	7.53	98.1	4.17
Shikimic acid	95.7	1.68	97.0	2.09	96.0	0.833	92.0	2.30
Glyceric acid	99.4	2.26	96.1	2.09	101	0.685	99.3	1.63
beta-Hydroxybutyric acid	100	1.22	99.2	2.42	103	1.47	103	0.896
Lactic acid	100	2.02	101	1.66	102	1.42	104	1.05
HPHPA	98.8	1.40	98.7	2.31	108	2.97	102	0.980
Propionic acid	98.5	3.33	92.5	1.80	100	2.59	101	2.43
5-Hydroxyindoleacetic acid	108	1.60	91.9	7.72	104	0.278	91.2	2.53
para-Hydroxyphenylacetic acid	105	2.60	104	2.69	99.5	5.32	100	2.30

Malic acid	97.0	3.23	99.9	1.33	102	2.08	103	0.900
Butyric acid	102	4.42	101	6.68	107	4.86	101	1.43
Hippuric acid	94.1	3.54	97.3	1.71	101	2.50	102	1.49
Succinic acid	98.9	1.42	98.4	1.77	101	2.17	100	1.95
Glutaric acid	93.5	3.75	98.6	3.41	103	2.55	105	2.53
Methylmalonic acid	95.2	2.22	103	2.72	96.3	2.16	103	5.86
Fumaric acid	98.9	6.18	96.5	3.45	102	2.17	104	1.42
Valeric acid	97.1	6.84	97.7	2.07	111	2.93	91.0	5.41
Benzoic acid	94.4	0.847	95.1	3.56	90.5	2.70	93.0	5.60
Oxalic acid	89.7	4.16	103	3.28	96.0	1.44	106	3.41
Indole acetic acid	97.5	6.54	95.2	7.69	103	5.96	90.3	7.09
Oxaloacetic acid	96.1	1.57	93.3	2.74	105	3.45	97.6	3.57
Salicylic acid	107	5.01	114	2.69	114	3.73	115	5.84
Citric acid	93.3	5.71	94.1	4.68	96.1	2.50	101	2.46
Abscisic acid	93.5	4.55	102	6.59	109	1.53	106	3.03
Aconitic acid	93.5	4.55	112	4.55	109	1.53	117	1.37
Jasmonic acid	83.7	4.02	98.7	5.64	88.8	1.56	99.7	3.24
Pyruvic acid	104	3.16	104	2.73	108	0.369	112	1.64
alpha-Ketoglutaric acid	102	0.781	101	1.99	107	0.749	107	2.07

High			
Intra-day		Inter-day	
Accuracy (%)	CV (%)	Accuracy (%)	CV (%)
101	4.61	101	3.01
96.3	3.90	97.5	3.25
105	1.28	97.9	5.14
96.6	0.856	96.7	4.91
93.6	5.12	102	3.72
96.9	2.07	101	2.44
94.2	4.24	97.2	5.90
88.3	3.94	96.8	3.37
103	2.35	98.9	4.68
96.7	9.90	97.8	6.96
96.3	4.73	98.0	8.34
98.1	4.55	99.0	4.73
87.2	1.64	95.8	5.35
94.8	5.99	93.7	9.67
91.7	5.52	96.8	8.23
95.8	0.753	91.8	12.7
97.4	7.45	96.8	3.76
93.8	10.2	92.6	10.6
101	4.35	98.0	4.19
103	4.08	101	0.649
96.0	4.88	98.9	3.98
91.5	2.40	98.4	3.36
98.9	5.93	96.9	5.10
103	4.14	97.5	6.27

100	4.34	101	5.28
91.7	3.77	95.2	9.01
93.0	0.845	94.5	1.64
103	2.45	92.5	1.70
106	5.33	98.6	3.25
94.3	3.95	97.1	3.99
87.6	3.15	93.8	6.46
92.5	8.28	94.8	5.07
90.9	3.17	94.8	3.24
98.2	2.34	92.0	9.88
97.7	0.402	91.8	9.03
98.1	3.37	95.9	5.15
100	3.75	99.9	8.66
106	5.52	99.5	4.93
95.2	7.02	94.1	1.98
95.2	4.87	101	3.12
104	1.93	100	3.46
100	5.06	101	2.65
103	2.42	101	1.95
97.2	1.83	100	3.61
94.8	3.24	98.1	3.68
96.1	2.44	96.3	6.55
92.0	5.86	102	5.80
96.3	9.00	91.2	2.69
101	2.65	98.4	0.704
105	2.02	101	0.977
101	0.990	99.7	0.301
97.6	0.271	101	1.54
104	3.38	99.5	3.30
102	5.09	100	0.838
96.1	5.82	99.0	1.66
95.8	1.45	97.5	2.77

104	0.223	102	2.67
104	2.07	98.6	2.07
101	1.31	99.5	0.506
102	2.21	99.5	0.870
101	7.14	94.5	5.78
101	8.09	95.6	5.04
103	3.42	102	0.564
95.0	2.59	93.9	1.77
92.0	2.07	94.9	1.32
112	1.44	111	1.45
108	3.75	100	4.25
105	5.41	102	1.97
106	4.34	113	3.02
101	0.990	102	1.49
101	6.51	95.3	2.56
101	6.51	111	2.39
92.8	3.37	93.1	4.41
99.4	2.12	97.1	1.37
102	2.32	98.5	2.41
