# Supplementary material for: A Comprehensive Targeted Metabolomics Assay for Crop Plant Sample Analysis
Source: Metabolites. 2021 May 11;11(5):303. doi: 10.3390/metabo11050303 (PMC8151637; doi:10.3390/metabo11050303)
Supplement: Supplementary file 1 [file metabolites-11-00303-s001.zip › Supplementary Table S5.pdf]

Supplementary Table S5. Recovery Performance in Spiked NIST® SRM® 1575A Pi[illegible]		
**Analyte**	**Recovery (%)**	
	**Low**	**Mid**
Creatinine	104	101
Glycine	103	106
Alanine	109	101
Serine	115	103
Histamine	108	114
Proline	107	112
Valine	109	106
Threonine	90.2	84.5
Phenylethylamine	102	109
Taurine	96.4	102
Putrescine	105	118
trans-Hydroxyproline	117	108
Leucine	103	94.0
Isoleucine	97.5	97.3
Asparagine	97.1	98.2
Aspartic acid	95.7	96.8
Glutamine	108	101
Glutamic acid	113	107
Methionine	100	101
Dopamine	97.9	95.8
Histidine	105	102
Phenylalanine	101	98.1
Arginine	112	104
Citrulline	110	106
Serotonin	100	102
Tyrosine	99.4	89.0
DOPA	118	108
Asymmetric dimethylarginine	109	96.4
Total dimethylarginine	92.8	100
Tryptophan	108	106
Kynurenine	105	99.0
Carnosine	86.3	86.6
Nitro-Tyrosine	91.5	88.0
Ornithine	93.8	92.0
Lysine	94.8	91.9
Spermidine	111	112
Spermine	113	104

Sarcosine	104	108
Diacetylspermine	106	94.9
Tyramine	108	100
Creatine	105	103
Betaine	95.6	109
Choline	100	114
Trimethylamine N-oxide	102	102
Methylhistidine	101	92.7
Proline-Betaine	117	103
Zeatin	116	98.2
Homoserine	98.8	98.4
Shikimic acid	103	103
Glyceric acid	94.4	95.6
beta-Hydroxybutyric acid	107	106
Lactic acid	105	103
HPHPA	104	108
Propionic acid	119	103
5-Hydroxyindoleacetic acid	102	114
para-Hydroxyphenylacetic acid	96.0	93.4
Malic acid	113	101
Butyric acid	106	104
Hippuric acid	103	102
Succinic acid	108	103
Glutaric acid	104	100
Methylmalonic acid	110	97.5
Fumaric acid	102	101
Valeric acid	115	94.5
Benzoic acid	103	96.7
Oxalic acid	104	116
Indole acetic acid	100	101
Oxaloacetic acid	94.3	93.3
Salicylic acid	114	107
Citric acid	111	111
Abscisic acid	105	92.7
Aconitic acid	94.1	96.4
Jasmonic acid	91.7	93.8
Pyruvic acid	114	101
alpha-Ketoglutaric acid	109	110

ne Needle Extracts
**High**
102
110
107
102
103
103
107
92.8
109
99.3
113
98.0
90.5
94.7
99.1
90.6
100
103
108
106
110
108
101
99.2
106
95.9
108
102
101
105
96.3
92.1
97.9
100
101
114
116

101
101
102
105
107
105
99.2
100
96.5
94.5
109
91.9
108
102
95.0
104
102
110
98.1
108
106
102
104
101
103
101
91.6
91.1
106
98.8
91.5
94.2
110
96.7
85.3
92.1
90.1
90.3
