# Supplementary material for: A Comprehensive Targeted Metabolomics Assay for Crop Plant Sample Analysis
Source: Metabolites. 2021 May 11;11(5):303. doi: 10.3390/metabo11050303 (PMC8151637; doi:10.3390/metabo11050303)
Supplement: Supplementary file 1 [file metabolites-11-00303-s001.zip › Supplementary Table S6.pdf]

Supplementary Table 6. Validation Performance of Analytes Analyzed *vi*			
**Analyte**	**Full Name**	**LOD (μM)**	**Accuracy (%)**
C0	Carnitine	0.222	92.6
C2	Acetylcarnitine	0.135	106
C3:1	Propenoylcarnitine	0.0256	/
C3	Propionylcarnitine	0.0824	91.9
C4:1	Butenylcarnitine	0.0240	/
C4	Butyrylcarnitine	0.0486	103
C3OH	Hydroxypropionylcarnitine	0.0337	/
C5:1	Tiglylcarnitine	0.0305	/
C5	Valerylcarnitine	0.0256	111
C4OH	Hydroxybutyrylcarnitine	0.0237	/
C6:1	Hexenoylcarnitine	0.0247	/
C6	Hexanoylcarnitine	0.0929	92.4
C5OH	Hydroxyvalerylcarnitine	0.0341	/
C5:1DC	Glutaconylcarnitin	0.0250	/
C5DC	Glutarylcarnitine	0.0191	/
C8	Octanoylcarnitine	0.0316	91.8
C5MDC	Methylglutarylcarnitine	0.0218	/
C9	Nonaylcarnitine	0.0110	/
C7DC	Pimelylcarnitine	0.0200	/
C10:2	Decadienylcarnitine	0.0490	/
C10:1	Decenoylcarnitine	0.118	/
C10	Decanoylcarnitine	0.0585	96.7
C12:1	Dodecenoylcarnitine	0.0786	/
C12	Dodecanoylcarnitine	0.0443	107
C14:2	Tetradecadienylcarnitine	0.0519	/
C14:1	Tetradecenoylcarnitine	0.0716	/
C14	Tetradecanoylcarnitine	0.0320	104
C12DC	Dodecanedioylcarnitine	0.0148	/
C14:2OH	Hydroxytetradecadienylcarnitine	0.0542	/
C14:1OH	Hydroxytetradecenoylcarnitine	0.0560	/
C16:2	Hexadecadienylcarnitine	0.0463	/
C16:1	Hexadecenoylcarnitine	0.0201	/
C16	Hexadecanoylcarnitine	0.0265	96.5
C16:2OH	Hydroxyhexadecadienylcarnitine	0.0368	/
C16:1OH	Hydroxyhexadecenoylcarnitine	0.0491	/
C16OH	Hydroxyhexadecanoylcarnitine	0.0426	/
C18:2	Octadecadienylcarnitine	0.0581	/
C18:1	Octadecenoylcarnitine	0.0411	/
C18	Octadecanoylcarnitine	0.0237	104

C18:1OH	Hydroxyoctadecenoylcarnitine	0.0568	/
Hexose	Hexose	18.5	96.3
LysoPC a C14:0	LysoPhosphatidylcholine acyl C14:0	0.194	/
LysoPC a C16:1	LysoPhosphatidylcholine acyl C16:1	0.0191	/
LysoPC a C16:0	LysoPhosphatidylcholine acyl C16:0	0.0474	/
LysoPC a C17:0	LysoPhosphatidylcholine acyl C17:0	0.152	/
LysoPC a C18:2	LysoPhosphatidylcholine acyl C18:2	0.0258	/
LysoPC a C18:1	LysoPhosphatidylcholine acyl C18:1	0.0541	108
LysoPC a C18:0	LysoPhosphatidylcholine acyl C18:0	0.274	105
LysoPC a C20:4	LysoPhosphatidylcholine acyl C20:4	0.0179	/
LysoPC a C20:3	LysoPhosphatidylcholine acyl C20:3	0.0303	/
LysoPC a C24:0	LysoPhosphatidylcholine acyl C24:0	0.128	/
LysoPC a C26:1	LysoPhosphatidylcholine acyl C26:1	0.0320	/
LysoPC a C26:0	LysoPhosphatidylcholine acyl C26:0	0.0269	/
LysoPC a C28:1	LysoPhosphatidylcholine acyl C28:1	0.0170	/
LysoPC a C28:0	LysoPhosphatidylcholine acyl C28:0	0.0765	/
PC aa C24:0	Phosphatidylcholine diacyl C24:0	0.0214	/
PC aa C26:0	Phosphatidylcholine diacyl C26:0	0.267	/
PC aa C28:1	Phosphatidylcholine diacyl C28:1	0.0488	/
PC ae C30:2	Phosphatidylcholine diacyl C30:2	0.0163	/
PC ae C30:1	Phosphatidylcholine diacyl C30:1	0.0193	/
PC ae C30:0	Phosphatidylcholine diacyl C30:0	0.0336	/
PC aa C30:2	Phosphatidylcholine diacyl C30:2	0.0403	/
PC aa C30:0	Phosphatidylcholine diacyl C30:0	0.0644	/
PC ae C32:2	Phosphatidylcholine acyl-alkyl C32:2	0.0139	/
PC ae C32:1	Phosphatidylcholine acyl-alkyl C32:1	0.0243	/
PC aa C32:3	Phosphatidylcholine diacyl C32:3	0.00767	/
PC aa C32:2	Phosphatidylcholine diacyl C32:2	0.0652	/
PC aa C32:1	Phosphatidylcholine diacyl C32:1	0.108	/
PC aa C32:0	Phosphatidylcholine diacyl C32:0	0.135	/
PC ae C34:3	Phosphatidylcholine acyl-alkyl C34:3	0.0513	/
PC ae C34:2	Phosphatidylcholine acyl-alkyl C34:2	0.0466	/
PC ae C34:1	Phosphatidylcholine acyl-alkyl C34:1	0.0810	/
PC ae C34:0	Phosphatidylcholine acyl-alkyl C34:0	0.0229	/
PC aa C34:4	Phosphatidylcholine diacyl C34:4	0.00953	/
PC aa C34:3	Phosphatidylcholine diacyl C34:3	0.0847	/
PC aa C34:2	Phosphatidylcholine diacyl C34:2	0.807	/
PC aa C34:1	Phosphatidylcholine diacyl C34:1	0.549	/
PC ae C36:5	Phosphatidylcholine acyl-alkyl C36:5	0.0775	/
PC ae C36:4	Phosphatidylcholine acyl-alkyl C36:4	0.0780	/
PC ae C36:3	Phosphatidylcholine acyl-alkyl C36:3	0.0489	/
PC ae C36:2	Phosphatidylcholine acyl-alkyl C36:2	0.106	/
PC ae C36:1	Phosphatidylcholine acyl-alkyl C36:1	0.115	/

PC ae C36:0	Phosphatidylcholine acyl-alkyl C36:0	0.0194	/
PC aa C36:6	Phosphatidylcholine diacyl C36:6	0.156	/
PC aa C36:5	Phosphatidylcholine diacyl C36:5	0.0910	/
PC aa C36:4	Phosphatidylcholine diacyl C36:4	0.805	/
PC aa C36:3	Phosphatidylcholine diacyl C36:3	0.582	/
PC aa C36:2	Phosphatidylcholine diacyl C36:2	1.18	/
PC aa C36:1	Phosphatidylcholine diacyl C36:1	0.472	/
PC aa C36:0	Phosphatidylcholine diacyl C36:0	0.0898	104
PC ae C38:6	Phosphatidylcholine acyl-alkyl C38:6	0.0552	/
PC ae C38:5	Phosphatidylcholine acyl-alkyl C38:5	0.181	/
PC ae C38:4	Phosphatidylcholine acyl-alkyl C38:4	0.109	/
PC ae C38:3	Phosphatidylcholine acyl-alkyl C38:3	0.0651	/
PC ae C38:2	Phosphatidylcholine acyl-alkyl C38:2	0.0653	/
PC ae C38:1	Phosphatidylcholine acyl-alkyl C38:1	0.0773	/
PC ae C38:0	Phosphatidylcholine acyl-alkyl C38:0	0.0227	/
PC aa C38:6	Phosphatidylcholine diacyl C38:6	0.0365	/
PC aa C38:5	Phosphatidylcholine diacyl C38:5	0.289	/
PC aa C38:4	Phosphatidylcholine diacyl C38:4	0.362	/
PC aa C38:3	Phosphatidylcholine diacyl C38:3	0.351	/
PC aa C38:1	Phosphatidylcholine diacyl C38:1	0.0706	/
PC aa C38:0	Phosphatidylcholine diacyl C38:0	0.0511	/
PC ae C40:6	Phosphatidylcholine acyl-alkyl C40:6	0.0686	/
PC ae C40:5	Phosphatidylcholine acyl-alkyl C40:5	0.0389	/
PC ae C40:4	Phosphatidylcholine acyl-alkyl C40:4	0.0145	/
PC ae C40:3	Phosphatidylcholine acyl-alkyl C40:3	0.0197	/
PC ae C40:2	Phosphatidylcholine acyl-alkyl C40:2	0.0197	/
PC ae C40:1	Phosphatidylcholine acyl-alkyl C40:1	0.0403	/
PC aa C40:6	Phosphatidylcholine diacyl C40:6	0.0586	/
PC aa C40:5	Phosphatidylcholine diacyl C40:5	0.0649	/
PC aa C40:4	Phosphatidylcholine diacyl C40:4	0.0338	/
PC aa C40:3	Phosphatidylcholine diacyl C40:3	0.0178	/
PC aa C40:2	Phosphatidylcholine diacyl C40:2	0.0120	/
PC aa C40:1	Phosphatidylcholine diacyl C40:1	0.125	/
PC ae C42:5	Phosphatidylcholine acyl-alkyl C42:5	0.0800	/
PC ae C42:4	Phosphatidylcholine acyl-alkyl C42:4	0.0108	/
PC ae C42:3	Phosphatidylcholine acyl-alkyl C42:3	0.0114	/
PC ae C42:2	Phosphatidylcholine acyl-alkyl C42:2	0.00852	/
PC ae C42:1	Phosphatidylcholine acyl-alkyl C42:1	0.0148	/
PC ae C42:0	Phosphatidylcholine acyl-alkyl C42:0	0.0686	/
PC aa C42:6	Phosphatidylcholine diacyl C42:6	0.0312	/
PC aa C42:5	Phosphatidylcholine diacyl C42:5	0.00799	/
PC aa C42:4	Phosphatidylcholine diacyl C42:4	0.00988	/
PC aa C42:2	Phosphatidylcholine diacyl C42:2	0.00831	/

PC aa C42:1	Phosphatidylcholine diacyl C42:1	0.00750	/
PC aa C42:0	Phosphatidylcholine diacyl C42:0	0.0179	/
PC ae C44:6	Phosphatidylcholine acyl-alkyl C44:6	0.0166	/
PC ae C44:5	Phosphatidylcholine acyl-alkyl C44:5	0.0240	/
PC ae C44:4	Phosphatidylcholine acyl-alkyl C44:4	0.00591	/
PC ae C44:3	Phosphatidylcholine acyl-alkyl C44:3	0.00384	/

a **DFI-MS/MS**	
**CV (%)**	**Recovery (%)**
4.28	98.5
8.14	101
/	/
3.28	95.6
/	/
5.66	108
/	/
/	/
9.21	108
/	/
/	/
6.97	94.2
/	/
/	/
/	/
4.44	109
/	/
/	/
/	/
/	/
/	/
6.82	93.6
/	/
7.31	111
/	/
/	/
6.27	105
/	/
/	/
/	/
/	/
/	/
5.55	103
/	/
/	/
/	/
/	/
/	/
7.68	96.8

/	/
2.91	103
/	/
/	/
/	/
/	/
/	/
7.63	106
5.17	104
/	/
/	/
/	/
/	/
/	/
/	/
/	/
/	/
/	/
/	/
/	/
/	/
/	/
/	/
/	/
/	/
/	/
/	/
/	/
/	/
/	/
/	/
/	/
/	/
/	/
/	/
/	/
/	/
/	/
/	/
/	/
/	/
/	/
/	/

/	/
/	/
/	/
/	/
/	/
/	/
/	/
6.22	101
/	/
/	/
/	/
/	/
/	/
/	/
/	/
/	/
/	/
/	/
/	/
/	/
/	/
/	/
/	/
/	/
/	/
/	/
/	/
/	/
/	/
/	/
/	/
/	/
/	/
/	/
/	/
/	/
/	/
/	/
/	/
/	/
/	/
/	/
/	/

/	/
/	/
/	/
/	/
/	/
/	/
