# Supplementary material for: A Comprehensive Targeted Metabolomics Assay for Crop Plant Sample Analysis
Source: Metabolites. 2021 May 11;11(5):303. doi: 10.3390/metabo11050303 (PMC8151637; doi:10.3390/metabo11050303)
Supplement: Supplementary file 1 [file metabolites-11-00303-s001.zip › Supplementary Table S7.pdf]

| Supplementary Table S7. Detected | |
|---|---|
| **Analyte** | |
| | **NIST® SRM® 1575a pine needle** |
| Creatinine | *ND |
| Glycine | 0.409 ± 0.00150 |
| Alanine | 0.569 ± 0.0165 |
| Serine | 0.885 ± 0.00900 |
| Histamine | ND |
| Proline | 0.591 ± 0.00301 |
| Valine | 0.224 ± 0.00345 |
| Threonine | 0.426 ± 0.00300 |
| Phenylethylamine | 0.00288 ± 0.000675 |
| Taurine | ND |
| Putrescine | 0.191 ± 0.00330 |
| trans-Hydroxyproline | 0.0132 ± 0.000449 |
| Leucine | 0.258 ± 0.00645 |
| Isoleucine | 0.0353 ± 0.000751 |
| Asparagine | 0.259 ± 0.0123 |
| Aspartic acid | 2.34 ± 0.0375 |
| Glutamine | 8.71 ± 0.0300 |
| Glutamic acid | 4.08 ± 0.0601 |
| Methionine | 0.0347 ± 0.000149 |
| Dopamine | 0.0624 ± 0.00181 |
| Histidine | 0.365 ± 0.00749 |
| Phenylalanine | 0.148 ± 0.000298 |
| Arginine | 52.1 ± 0.751 |
| Citrulline | ND |
| Serotonin | ND |
| Tyrosine | 0.0426 ± 0.000897 |
| DOPA | ND |
| Asymmetric dimethylarginine | 0.0128 ± 0.000135 |
| Total dimethylarginine | 0.0204 ± 0.000165 |
| Tryptophan | 0.0866 ± 0.000748 |
| Kynurenine | ND |
| Carnosine | ND |
| Nitro-Tyrosine | ND |
| Ornithine | 5.36 ± 0.225 |
| Lysine | 4.28 ± 0.105 |
| Spermidine | 0.0375 ± 0.000896 |
| Spermine | 0.0159 ± 0.000751 |

| | |
|---|---|
| Sarcosine | ND |
| Diacetylspermine | ND |
| Tyramine | 0.00462 ± 0.000314 |
| Creatine | ND |
| Betaine | 0.0407 ± 0.000449 |
| Choline | 3.36 ± 0.0304 |
| Trimethylamine N-oxide | ND |
| Methylhistidine | ND |
| Proline-Betaine | 0.0684 ± 0.00912 |
| Zeatin | ND |
| Homoserine | ND |
| Shikimic acid | 2.14 ± 0.0149 |
| Glyceric acid | 2.01 ± 0.0592 |
| beta-Hydroxybutyric acid | 0.0362 ± 0.00105 |
| Lactic acid | ND |
| HPHPA | ND |
| Propionic acid | 0.429 ± 0.0187 |
| 5-Hydroxyindoleacetic acid | ND |
| para-Hydroxyphenylacetic acid | 0.00123 ± 0.000225 |
| Malic acid | 5.93 ± 0.0151 |
| Butyric acid | 0.134 ± 0.00421 |
| Hippuric acid | ND |
| Succinic acid | 0.416 ± 0.00152 |
| Glutaric acid | 0.241 ± 0.00405 |
| Methylmalonic acid | ND |
| Fumaric acid | 0.122 ± 0.000146 |
| Valeric acid | 0.0392 ± 0.000746 |
| Benzoic acid | ND |
| Oxalic acid | 18.5 ± 0.135 |
| Indole acetic acid | ND |
| Oxaloacetic acid | ND |
| Salicylic acid | ND |
| Citric acid | 12.5 ± 0.0147 |
| Abscisic acid | ND |
| Aconitic acid | 0.0231 ± 0.000585 |
| Jasmonic acid | ND |
| Pyruvic acid | ND |
| alpha-Ketoglutaric acid | 0.0572 ± 0.000303 |
| Hexose | 319 ± 10.1 |
| C0 | 0.0434 ± 0.000538 |
| C2 | ND |
| C3:1 | ND |
| C3 | ND |

| | |
|---|---|
| C4:1 | ND |
| C4 | ND |
| C3OH | ND |
| C5:1 | ND |
| C5 | ND |
| C4OH | ND |
| C6:1 | ND |
| C6 | ND |
| C5OH | ND |
| C5:1DC | ND |
| C5DC | ND |
| C8 | ND |
| C5MDC | ND |
| C9 | ND |
| C7DC | ND |
| C10:2 | ND |
| C10:1 | ND |
| C10 | ND |
| C12:1 | ND |
| C12 | ND |
| C14:2 | ND |
| C14:1 | ND |
| C14 | ND |
| C12DC | ND |
| C14:2OH | ND |
| C14:1OH | ND |
| C16:2 | ND |
| C16:1 | ND |
| C16 | ND |
| C16:2OH | ND |
| C16:1OH | ND |
| C16OH | ND |
| C18:2 | ND |
| C18:1 | ND |
| C18 | ND |
| C18:1OH | ND |
| LysoPC a C14:0 | 0.00674 ± 0.000109 |
| LysoPC a C16:1 | ND |
| LysoPC a C16:0 | 0.0313 ± 0.000137 |
| LysoPC a C17:0 | ND |
| LysoPC a C18:2 | 0.0354 ± 0.000121 |
| LysoPC a C18:1 | 0.0235 ± 0.000337 |
| LysoPC a C18:0 | 0.00319 ± 0.000144 |

| | |
|---|---|
| LysoPC a C20:4 | 0.00351 ± 0.000152 |
| LysoPC a C20:3 | 0.0302 ± 0.000228 |
| LysoPC a C24:0 | ND |
| LysoPC a C26:1 | ND |
| LysoPC a C26:0 | 0.00325 ± 0.000301 |
| LysoPC a C28:1 | 0.00608 ± 0.000109 |
| LysoPC a C28:0 | 0.00648 ± 0.000208 |
| PC aa C24:0 | 0.000686 ± 0.0000191 |
| PC aa C26:0 | 0.00789 ± 0.00134 |
| PC aa C28:1 | 0.000827 ± 0.000124 |
| PC ae C30:2 | ND |
| PC ae C30:1 | ND |
| PC ae C30:0 | 0.00276 ± 0.000876 |
| PC aa C30:2 | 0.000617 ± 0.000105 |
| PC aa C30:0 | 0.00171 ± 0.000648 |
| PC ae C32:2 | 0.000281 ± 0.000111 |
| PC ae C32:1 | ND |
| PC aa C32:3 | 0.000701 ± 0.000263 |
| PC aa C32:2 | ND |
| PC aa C32:1 | ND |
| PC aa C32:0 | ND |
| PC ae C34:3 | ND |
| PC ae C34:2 | ND |
| PC ae C34:1 | ND |
| PC ae C34:0 | ND |
| PC aa C34:4 | 0.00538 ± 0.000978 |
| PC aa C34:3 | 0.0464 ± 0.00394 |
| PC aa C34:2 | 0.101 ± 0.0652 |
| PC aa C34:1 | 0.0669 ± 0.00189 |
| PC ae C36:5 | ND |
| PC ae C36:4 | ND |
| PC ae C36:3 | 0.00376 ± 0.00153 |
| PC ae C36:2 | 0.00874 ± 0.00416 |
| PC ae C36:1 | 0.00839 ± 0.00119 |
| PC ae C36:0 | 0.00499 ± 0.0000128 |
| PC aa C36:6 | 0.0137 ± 0.0003397 |
| PC aa C36:5 | 0.0246 ± 0.00861 |
| PC aa C36:4 | 0.0643 ± 0.00977 |
| PC aa C36:3 | 0.0949 ± 0.0134 |
| PC aa C36:2 | 0.0585 ± 0.00989 |
| PC aa C36:1 | 0.0113 ± 0.00888 |
| PC aa C36:0 | 0.00348 ± 0.000157 |
| PC ae C38:6 | 0.00154 ± 0.00104 |

| | |
|---|---|
| PC ae C38:5 | 0.00104 ± 0.000593 |
| PC ae C38:4 | 0.00238 ± 0.000999 |
| PC ae C38:3 | 0.00509 ± 0.00167 |
| PC ae C38:2 | 0.00434 ± 0.00118 |
| PC ae C38:1 | 0.00216 ± 0.000784 |
| PC ae C38:0 | ND |
| PC aa C38:6 | 0.0160 ± 0.000146 |
| PC aa C38:5 | 0.0173 ± 0.00698 |
| PC aa C38:4 | 0.0187 ± 0.00886 |
| PC aa C38:3 | 0.00811 ± 0.00159 |
| PC aa C38:1 | 0.00173 ± 0.000666 |
| PC aa C38:0 | ND |
| PC ae C40:6 | ND |
| PC ae C40:5 | ND |
| PC ae C40:4 | ND |
| PC ae C40:3 | ND |
| PC ae C40:2 | ND |
| PC ae C40:1 | ND |
| PC aa C40:6 | ND |
| PC aa C40:5 | ND |
| PC aa C40:4 | ND |
| PC aa C40:3 | ND |
| PC aa C40:2 | ND |
| PC aa C40:1 | ND |
| PC ae C42:5 | ND |
| PC ae C42:4 | ND |
| PC ae C42:3 | ND |
| PC ae C42:2 | ND |
| PC ae C42:1 | ND |
| PC ae C42:0 | ND |
| PC aa C42:6 | ND |
| PC aa C42:5 | ND |
| PC aa C42:4 | ND |
| PC aa C42:2 | ND |
| PC aa C42:1 | ND |
| PC aa C42:0 | ND |
| PC ae C44:6 | ND |
| PC ae C44:5 | ND |
| PC ae C44:4 | ND |
| PC ae C44:3 | ND |

*ND: Not Detected

**and Quantified Plant Metabolite Concentrations in Different Samples**

**Concentrations (µmol per gram)**

| Canola Root Samples | Commercial Cannabis Strains |
|---|---|
| ND | ND |
| 0.942 ± 0.787 | 1.44 ± 0.579 |
| 4.56 ± 2.82 | 4.27 ± 0.867 |
| 4.79 ± 5.34 | 4.59 ± 1.07 |
| 0.00636 ± 0.0229 | 0.00413 ± 0.00246 |
| 22.9 ± 33.4 | 8.91 ± 7.81 |
| 1.67 ± 1.29 | 2.99 ± 1.23 |
| 2.97 ± 2.75 | 1.77 ± 0.457 |
| 0.0216 ± 0.0191 | 0.00177 ± 0.00202 |
| 0.00906 ± 0.00469 | 0.0201 ± 0.0126 |
| 0.101 ± 0.0897 | 0.0126 ± 0.00368 |
| 0.0312 ± 0.0327 | 0.0256 ± 0.00583 |
| 1.14 ± 0.897 | 1.37 ± 0.606 |
| 0.745 ± 0.678 | 1.97 ± 1.19 |
| 3.04 ± 4.02 | 156 ± 69.8 |
| 2.79 ± 4.58 | 7.79 ± 3.60 |
| 14.6 ± 12.9 | 22.3 ± 12.7 |
| 8.31 ± 6.05 | 2.60 ± 1.10 |
| 0.143 ± 0.201 | 0.0703 ± 0.0171 |
| ND | ND |
| 0.371 ± 0.343 | 4.05 ± 1.81 |
| 0.478 ± 0.484 | 1.21 ± 0.651 |
| 0.491 ± 0.411 | 30.1 ± 8.90 |
| 0.122 ± 0.158 | 0.291 ± 0.114 |
| 0.0374 ± 0.0846 | 0.0958 ± 0.0291 |
| 0.276 ± 0.187 | 0.384 ± 0.113 |
| ND | ND |
| 0.0256 ± 0.0274 | 0.0159 ± 0.0109 |
| 0.0377 ± 0.0370 | 0.0351 ± 0.00907 |
| 0.188 ± 0.225 | 1.85 ± 1.22 |
| 0.00167 ± 0.000986 | 0.00407 ± 0.00290 |
| ND | ND |
| ND | ND |
| 0.0743 ± 0.0792 | 0.250 ± 0.245 |
| 0.569 ± 0.562 | 1.62 ± 0.761 |
| 0.0331 ± 0.0402 | 0.0216 ± 0.0189 |
| 0.00332 ± 0.00478 | 0.00390 ± 0.00237 |

| | |
|---|---|
| ND | ND |
| ND | ND |
| 0.721 ± 1.14 | 0.0996 ± 0.0980 |
| ND | 0.0210 ± 0.00764 |
| 0.651 ± 0.458 | 0.0992 ± 0.0342 |
| 3.60 ± 2.44 | 10.33 ± 1.89 |
| ND | 0.0407 ± 0.00452 |
| 0.0172 ± 0.0125 | 0.0263 ± 0.0105 |
| 0.417 ± 0.0830 | 0.133 ± 0.108 |
| 0.198 ± 0.0712 | 0.0958 ± 0.0294 |
| 0.0116 ± 0.00525 | ND |
| 0.609 ± 1.03 | 0.009001 ± 0.00217 |
| 0.627 ± 0.741 | 1.92 ± 0.817 |
| 0.115 ± 0.381 | 0.0690 ± 0.0187 |
| 4.45 ± 7.09 | 0.273 ± 0.159 |
| ND | 0.000733 ± 0.0000194 |
| 0.0979 ± 0.0272 | 0.207 ± 0.106 |
| ND | ND |
| 0.00516 ± 0.00232 | 0.0126 ± 0.00397 |
| 18.2 ± 19.4 | 3.98 ± 1.97 |
| 0.0568 ± 0.0290 | 0.222 ± 0.123 |
| 0.00633 ± 0.00429 | 0.00741 ± 0.00526 |
| 0.685 ± 0.732 | 0.324 ± 0.103 |
| 0.0440 ± 0.0262 | 0.324 ± 0.178 |
| 0.00341 ± 0.00463 | 0.00230 ± 0.000524 |
| 0.340 ± 0.330 | 0.217 ± 0.214 |
| 0.0157 ± 0.00606 | ND |
| 0.0194 ± 0.0189 | 0.497 ± 0.228 |
| 1.86 ± 1.66 | 3.18 ± 6.28 |
| 0.0168 ± 0.00785 | ND |
| ND | 0.120 ± 0.0399 |
| ND | 0.117 ± 0.0517 |
| 12.5 ± 12.0 | 30.3 ± 16.8 |
| ND | 0.00583 ± 0.00378 |
| 0.195 ± 0.190 | 3.10 ± 2.34 |
| ND | 0.0683 ± 0.0349 |
| 0.674 ± 1.40 | 0.0290 ± 0.00598 |
| 1.71 ± 2.54 | 0.0127 ± 0.00750 |
| 62.7 ± 52.9 | 45.9 ± 47.8 |
| 0.0104 ± 0.00495 | 0.0276 ± 0.00934 |
| ND | ND |
| ND | ND |
| ND | ND |

| | |
|---|---|
| ND | ND |
| ND | ND |
| ND | ND |
| ND | ND |
| ND | ND |
| ND | ND |
| ND | ND |
| ND | ND |
| ND | ND |
| ND | ND |
| ND | ND |
| ND | ND |
| ND | ND |
| ND | ND |
| ND | ND |
| 0.00671 ± 0.00184 | ND |
| 0.00804 ± 0.00292 | ND |
| 0.00911 ± 0.00369 | ND |
| ND | ND |
| 0.00863 ± 0.00111 | ND |
| ND | ND |
| ND | ND |
| 0.00547 ± 0.00102 | ND |
| ND | ND |
| ND | ND |
| ND | ND |
| ND | ND |
| ND | ND |
| ND | ND |
| ND | ND |
| ND | ND |
| ND | ND |
| ND | ND |
| ND | ND |
| ND | ND |
| ND | ND |
| 0.00264 ± 0.000374 | ND |
| ND | ND |
| 0.00361 ± 0.00351 | 0.0123 ± 0.00371 |
| ND | ND |
| 0.00444 ± 0.00408 | 0.0146 ± 0.00735 |
| 0.000978 ± 0.000768 | 0.00334 ± 0.000997 |
| ND | 0.0152 ± 0.00453 |

| | |
|---|---|
| ND | ND |
| ND | ND |
| ND | ND |
| ND | ND |
| ND | ND |
| ND | ND |
| ND | ND |
| ND | ND |
| ND | ND |
| ND | ND |
| ND | ND |
| ND | ND |
| ND | ND |
| ND | ND |
| ND | ND |
| 0.00115 ± 0.00154 | ND |
| ND | ND |
| 0.00202 ± 0.00200 | ND |
| ND | ND |
| 0.00250 ± 0.00197 | ND |
| ND | ND |
| 0.00392 ± 0.00341 | ND |
| 0.00496 ± 0.00431 | ND |
| 0.00176 ± 0.00122 | ND |
| ND | ND |
| 0.00289 ± 0.00223 | ND |
| 0.178 ± 0.191 | ND |
| 0.305 ± 0.302 | ND |
| 0.0524 ± 0.0494 | ND |
| ND | ND |
| 0.00221 ± 0.00166 | ND |
| 0.00454 ± 0.00367 | ND |
| 0.00517 ± 0.00370 | ND |
| 0.00185 ± 0.00109 | ND |
| ND | 0.00173 ± 0.000912 |
| 0.0655 ± 0.0792 | 0.00191 ± 0.000340 |
| 0.188 ± 0.203 | ND |
| 0.251 ± 0.266 | 0.0211 ± 0.00982 |
| 0.0677 ± 0.0618 | 0.0388 ± 0.0121 |
| 0.0290 ± 0.0205 | 0.0268 ± 0.00854 |
| 0.00463 ± 0.00336 | ND |
| 0.00106 ± 0.000738 | ND |
| 0.00139 ± 0.00108 | ND |

| | |
|---|---|
| 0.00364 ± 0.00377 | ND |
| 0.00395 ± 0.00414 | ND |
| 0.00246 ± 0.00246 | ND |
| 0.000831 ± 0.000854 | ND |
| 0.00137 ± 0.00153 | ND |
| ND | ND |
| 0.00232 ± 0.00215 | 0.00159 ± 0.000833 |
| 0.00352 ± 0.00308 | 0.00622 ± 0.00111 |
| 0.00391 ± 0.00349 | 0.00429 ± 0.000711 |
| 0.00398 ± 0.00324 | ND |
| ND | ND |
| 0.00251 ± 0.000328 | ND |
| ND | ND |
| ND | ND |
| ND | ND |
| 0.00531 ± 0.00851 | ND |
| 0.00158 ± 0.00158 | ND |
| ND | ND |
| ND | ND |
| ND | ND |
| ND | ND |
| ND | ND |
| ND | ND |
| ND | ND |
| 0.00752 ± 0.0060766 | ND |
| 0.000888 ± 0.000991 | ND |
| 0.000862 ± 0.000788 | ND |
| ND | ND |
| ND | ND |
| ND | ND |
| ND | ND |
| ND | ND |
| 0.000856 ± 0.00141 | ND |
| 0.00226 ± 0.00279 | ND |
| ND | ND |
| ND | ND |
| ND | ND |
| ND | ND |
| ND | ND |
| ND | ND |

| |
|---|
| |
| |
| **Spruce & Pine Needles** |
| ND |
| 1.20 ± 0.949 |
| 0.817 ± 0.304 |
| 1.98 ± 0.753 |
| 0.00314 ± 0.000846 |
| 28.1 ± 19.4 |
| 0.420 ± 0.0883 |
| 0.800 ± 0.191 |
| 0.00401 ± 0.00149 |
| ND |
| 1.48 ± 0.428 |
| 0.0322 ± 0.0449 |
| 0.560 ± 0.199 |
| 0.351 ± 0.241 |
| 1.58 ± 1.50 |
| 6.64 ± 3.78 |
| 13.4 ± 7.48 |
| 12.8 ± 4.58 |
| 0.115 ± 0.0725 |
| 0.0219 ± 0.00167 |
| 1.02 ± 0.798 |
| 0.590 ± 0.202 |
| 65.0 ± 45.1 |
| 0.0122 ± 0.00121 |
| 0.0256 ± 0.00987 |
| 0.225 ± 0.124 |
| ND |
| 0.0134 ± 0.00656 |
| 0.0229 ± 0.00135 |
| 0.992 ± 0.873 |
| ND |
| ND |
| ND |
| 11.3 ± 10.7 |
| 18.6 ± 18.1 |
| 0.0277 ± 0.00789 |
| 0.00629 ± 0.0000964 |

| |
|---|
| 0.0389 ± 0.00452 |
| ND |
| 0.00576 ± 0.000684 |
| 0.00591 ± 0.00294 |
| 0.0633 ± 0.0446 |
| 4.55 ± 2.88 |
| ND |
| 0.0206 ± 0.00961 |
| 0.0346 ± 0.0123 |
| 0.0108 ± 0.00294 |
| 0.0382 ± 0.00516 |
| 57.1 ± 21.1 |
| 0.713 ± 0.284 |
| 0.0801 ± 0.0308 |
| 0.277 ± 0.189 |
| ND |
| 0.0962 ± 0.0351 |
| ND |
| ND |
| 10.2 ± 4.43 |
| 0.0599 ± 0.0271 |
| 0.0108 ± 0.00785 |
| 0.348 ± 0.262 |
| 0.00597 ± 0.00478 |
| ND |
| 0.0371 ± 0.0226 |
| 0.0298 ± 0.0109 |
| 0.0659 ± 0.0370 |
| 24.0 ± 15.4 |
| ND |
| ND |
| 0.0763 ± 0.0521 |
| 3.79 ± 2.93 |
| ND |
| 0.0150 ± 0.0131 |
| ND |
| ND |
| 0.255 ± 0.232 |
| 379 ± 101 |
| 0.0228 ± 0.0322 |
| ND |
| ND |
| ND |

| |
|---|
| ND |
| ND |
| ND |
| ND |
| ND |
| ND |
| ND |
| ND |
| ND |
| ND |
| ND |
| ND |
| ND |
| ND |
| ND |
| ND |
| ND |
| ND |
| ND |
| ND |
| ND |
| ND |
| ND |
| ND |
| ND |
| ND |
| ND |
| ND |
| ND |
| ND |
| ND |
| ND |
| ND |
| ND |
| ND |
| ND |
| 0.00448 ± 0.00160 |
| ND |
| 0.0471 ± 0.0273 |
| 0.00257 ± 0.00209 |
| 0.0308 ± 0.0240 |
| 0.0175 ± 0.0163 |
| 0.00541 ± 0.00305 |

| |
|---|
| ND |
| ND |
| ND |
| ND |
| ND |
| ND |
| ND |
| ND |
| ND |
| ND |
| ND |
| ND |
| ND |
| ND |
| ND |
| ND |
| ND |
| ND |
| ND |
| ND |
| ND |
| ND |
| 0.00319 ± 0.00174 |
| 0.00373 ± 0.00201 |
| 0.00203 ± 0.00172 |
| 0.0112 ± 0.00979 |
| 0.173 ± 0.0963 |
| 0.296 ± 0.139 |
| 0.145 ± 0.0681 |
| ND |
| 0.00238 ± 0.00175 |
| 0.0136 ± 0.00866 |
| 0.0280 ± 0.0207 |
| 0.0198 ± 0.0164 |
| ND |
| 0.0255 ± 0.0160 |
| 0.0849 ± 0.0457 |
| 0.170 ± 0.0826 |
| 0.269 ± 0.132 |
| 0.102 ± 0.0466 |
| 0.0274 ± 0.0120 |
| 0.00837 ± 0.00516 |
| 0.00276 ± 0.00245 |

| |
|---|
| 0.00428 ± 0.00415 |
| 0.0117 ± 0.0100 |
| 0.0225 ± 0.0164 |
| 0.0137 ± 0.0116 |
| 0.00463 ± 0.00381 |
| 0.00759 ± 0.00496 |
| 0.0413 ± 0.0259 |
| 0.0844 ± 0.0463 |
| 0.0576 ± 0.0295 |
| 0.0335 ± 0.0167 |
| 0.00677 ± 0.00454 |
| 0.00420 ± 0.00202 |
| ND |
| 0.00489 ± 0.00461 |
| 0.00638 ± 0.00581 |
| 0.00513 ± 0.00385 |
| 0.00315 ± 0.00227 |
| 0.00301 ± 0.00233 |
| 0.00769 ± 0.00430 |
| 0.00438 ± 0.00240 |
| ND |
| 0.00374 ± 0.00261 |
| ND |
| ND |
| 0.00504 ± 0.00145 |
| ND |
| ND |
| ND |
| ND |
| ND |
| ND |
| ND |
| ND |
| ND |
| ND |
| ND |
| ND |
| ND |
| ND |
| ND |
